# A Summary on Tuberculosis Vaccine Development—Where to Go?

**DOI:** 10.3390/jpm13030408

**Published:** 2023-02-24

**Authors:** Fan Jiang, Tiehui Sun, Peng Cheng, Jie Wang, Wenping Gong

**Affiliations:** 1 Tuberculosis Prevention and Control Key Laboratory/Beijing Key Laboratory of New Techniques of Tuberculosis Diagnosis and Treatment, Senior Department of Tuberculosis, The 8th Medical Center of PLA General Hospital, Beijing 100091, China; 2 The Second Brigade of Cadet, Basic Medical School, Air Force Military Medical University, Xi’an 710032, China; 3 Department of Gastroenterology, Zhang Jia Kou Fifth Hospital, Zhangjiakou 075051, China

**Keywords:** tuberculosis, vaccine, *Mycobacterium tuberculosis*, bibliometric analysis, immunity

## Abstract

Background: Tuberculosis (TB) is an old infectious disease caused by *Mycobacterium tuberculosis* infection. Vaccination is the most effective way to prevent and control TB. However, there is relatively little literature that systematically analyzes the progress of new TB vaccine research from a bibliometric perspective. This study was conducted to examine the development of TB vaccines over the past 20 years and to identify research priorities and directions for the future. Methods: The Science Citation Index Expanded (SCI-E) of the Web of Science Core Collection (WOSCC) database was selected to search the literature related to TB vaccines. The countries, institutions, authors, journals, references, and keywords of each publication were analyzed and visualized using the VOSviewer, CiteSpace, and Bibliometrix software. Furthermore, GraphPad Prism and Microsoft Excel 365 were also used for statistical analysis. Results: As of 20 October 2022, 7960 publications related to TB vaccines were identified with 288,478 citations. The United States of America (USA) accounted for the largest share (2658, 33.40%), followed by the United Kingdom (UK, 1301, 16.34%), and China (685, 8.6%). Regarding affiliations, the University of London had the most publications (427) and shared the highest H-index (76) with the Statens Serum Institut of Denmark. In terms of the number of articles for the journals and authors, the journal Vaccine ranked first with 629 articles. Professor Peter Anderssen has published the highest number of papers (160). The burst keywords and thematic maps analysis showed that future trends in TB vaccine development would focus on exploring the interaction mechanisms between *M. tuberculosis* and the host. Conclusion: The number of publications on TB vaccines has grown over the past two decades. Developed countries play a significant role in TB vaccine research, and developing countries are fast catching up. We believe that future research will be aimed at understanding the fine molecular mechanisms of host–pathogen interaction, leading to the development of better TB vaccines.

## 1. Introduction

Tuberculosis (TB) is an infectious disease caused by *Mycobacterium tuberculosis*. According to the World Health Organization’s (WHO) Global Tuberculosis Report 2022, there were 10.4 million new TB cases worldwide in 2021, with 87% of these new cases occurring in the 30 high-burden countries [1]. In particular, eight high-burden countries accounted for more than 66% of new TB cases, including India, China, Indonesia, Philippines, Pakistan, Nigeria, Bangladesh, and South Africa. Therefore, the prevention of TB is still a significant challenge. Bacille Calmette-Guerin (BCG) is the only vaccine approved for TB prevention. However, BCG vaccination has a defensive efficiency of 0% to 80% in adults [2,3,4,5].

A growing number of new TB vaccines have been developed to overcome these shortcomings of the BCG vaccine. Most of these new vaccines are in the preclinical stage, and more than 16 vaccines have been evaluated in clinical trials [6]. However, in the era of rapid information increase, many research results with important scientific innovation and application value are not known as soon as they are published. These excellent research results often take time to precipitate, researchers continue to cite, and then more people understand and recognize them. This is why Nobel laureates win their awards much later than their research achievements [7].

Fortunately, the rapid development of bioinformatics analysis has laid the foundation for using bibliometric analysis to investigate literature data worldwide. Bibliometrics is an interdisciplinary science that uses mathematical and statistical methods to analyze all knowledge carriers quantitatively [8]. It is a comprehensive knowledge system that integrates mathematics, statistics, and literature and pays attention to quantification. Bibliometrics analysis software and data visualization technology have been widely used to analyze the target research field qualitatively, provide substantial evidence for the influence of the research direction, and identify emerging research hotspots as well as potential research partners [8].

This study comprehensively investigated the number of publications, citations, H-index, the distribution of research institutions and authors, research hotspots, and future development trends in the field of TB vaccines by using bibliometric analysis, highlighting new perspectives and strategies for developing novel TB vaccines. To our knowledge, this is the first study to provide a comprehensive bibliometric analysis for TB vaccines.

## 2. Materials and Methods

### 2.1. Data Materials

In this study, the Science Citation Index Expanded (SCI-E) of the Web of Science Core Collection (WOSCC) database was selected as an eligible literature source for the following reasons: (1) SCI-E can provide the document pattern needed for bibliometrics analysis software such as CiteSpace and VOSviewer; (2) SCI-E database is the most authoritative and highest standard global database, which researchers widely use. The research on the TB vaccine originated from the BCG vaccine more than 100 years ago [2,9,10]. However, early publications on TB vaccines were minimal due to poor publishing technology and information technology, and the data recorded in the database did not gradually increase until the middle of the twentieth century. In addition, the upper limit of the number of articles analyzed by VOSviewer and CiteSpace software and SCI-E database citation reports is 10,000. Therefore, the literature published between 1995 to 2022 was included in this study.

### 2.2. Retrieval Strategies

**Figure 1:**
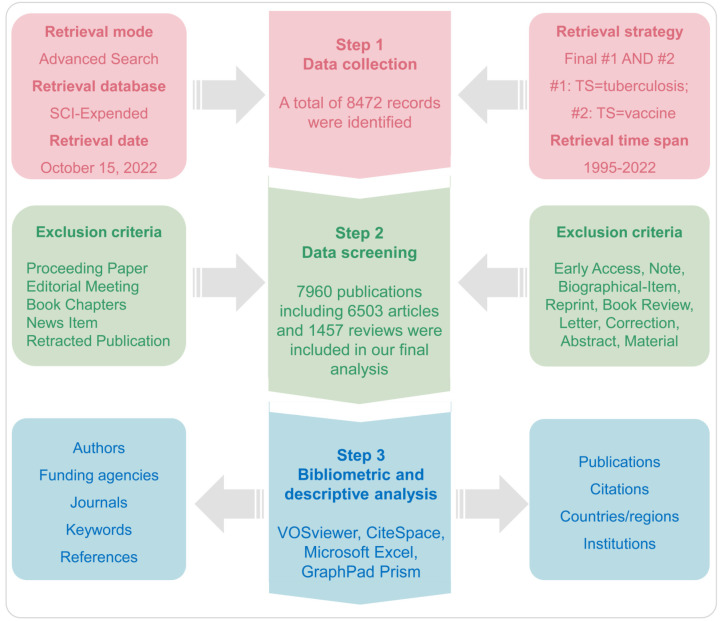
Flowchart of the literature search and selection process. A total of 8472 results were retrieved from the database. After excluding invalid articles, 7960 publications were eventually included, including “article” and 1457 “review” papers. Based on these documents, institutions conducting research, contributing authors, journals published in specific research areas, and countries or regions interested in the research field of vaccines for TB can be assessed.

The search terms in the SCI-E database were “#1 Thematic Suffix (TS)=Tuberculosis” and “#2 TS=vaccine”. Therefore, the search strategy was #1 AND #2. The inclusion criteria for publications were as follows: (1) The type of literature was “article” or “review”; (2) Literature was published between January 1995 and October 2022; (3) The topic search was “tuberculosis vaccines” or “TB vaccines”; (4) Papers were published in English. A total of 8472 publications were included, and 7960 ones were included after excluding 512 duplicate records, including 6503 “article” and 1457 “review” papers. Then, these 7960 bibliographic records were downloaded, including titles, author names, affiliations, abstracts, keywords, and citations. The literature screening process is shown in Figure 1.

### 2.3. Data Extraction

The data obtained from the SCI-E database were imported into Microsoft Excel 365 (Microsoft, Raymond, Washington, DC, USA) for further processing. This study mainly focused on the annual number of publications and citations, countries, journals, institutions, authors, citations, and keywords. The H-index and the cited frequency were evaluated using the citation report automatically generated by SCI-E. The H-index is calculated from h papers published by a scientist/country, each of which is cited at least h times [11,12]. This index is often used to assess the scientific research influence and productivity of researchers/countries.

### 2.4. Data Visualization and Analysis

In this study, CiteSpace and VOSviewer were used for the analysis. VOSviewer (version 1.6.18, Holland, downloaded from http://vosviewer.com, accessed on 15 October 2022) is a document knowledge visualization software based on the Visualization of Similarity (VOS) technology. VOSviewer was originally jointly developed by Ike and Waltman from Leiden University based on the JAVA platform. Features that the VOSviewer can analyze include co-authors, research institutions, countries, keywords, and co-cited references. Each node corresponds to a parameter in a network visual map, such as a country, institution, author, or keyword. There are three types of web maps that the VOSviewer can provide, including network visual maps, density visual maps, and overlay visual maps. The content of a map created, visualized, and explored using VOSviewer can include many projects. The meaning of a project is the object of interest. For example, a project can be a publication, a researcher, or a term. Maps usually contain only one type of project. For example, having a map containing both publications and terms is not common. In this study, we define the concept of a cluster as follows: when analyzing a project with several characteristics, we can divide it into small groups according to the project’s features, and these small groups are called clusters.

### 2.5. CiteSpace

CiteSpace (Version 6.1. R3, downloaded from https://sourceforge.net/projects/citespace/, accessed on 17 October 2022) is a computer program developed by Professor Chen based on the JAVA platform [13]. It is a highly influential visualization software that can obtain quantitative information and discover relevant developments and trends in specific scientific research fields. The web map generated by CiteSpace consists of nodes and links. Nodes usually represent the contribution of authors, countries, or institutions. The link describes the cooperative relationship between these nodes. Centrality is an important index to evaluate the importance of nodes in the network. The higher the node’s centrality, the more significant the impact of the node on the map. The burst analysis of references and keywords can identify the sharp increase in a particular type of scientific activity in a specific period. It can capture points of interest that will explode in a particular field of research in the future.

### 2.6. Ethics Statement

This study did not involve human or animal subjects. All data used for analysis were obtained from public databases. Therefore, ethical approval was not needed.

### 2.7. Statistical Analysis

Statistical analysis was performed using GraphPad Prism 9.5.1 (San Diego, CA, USA) and Microsoft Excel 365 (Microsoft Corporation, Shanghai, China). Bibliometric analysis was conducted by the VOSviewer (version 1.6.18, Leiden University, Leiden, The Netherlands), CiteSpace (version 6.1.R3, Drexel University, Philadelphia, PA, USA), and Bibliomtrix package of RStudio software 2022.07.2 (R version 4.2.1, download package Bibliomtrix 4.0.1).

## 3. Results

### 3.1. Number of Publication Outputs and Citations over the Years

**Figure 2:**
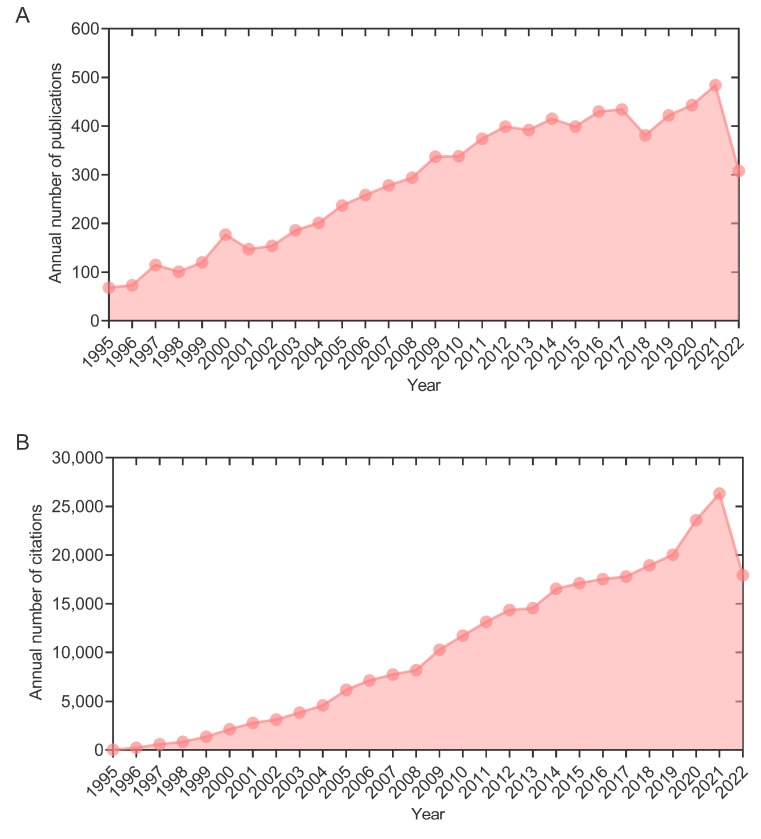
The annual number of publications and the total number of citations related to vaccines for TB from 1995 to 2022. The annual number of publications associated with TB vaccines (**A**) and their annual number of citations (**B**) increased gradually from 1995 to 2021. However, the number of publications and citations in 2022 was only counted up to October, so the number of publications and citations was lower than in 2021.

A total of 8472 records related to TB vaccines were retrieved from the SCI-E database from the last two decades, from January 1995 to October 2022. After excluding invalid records, 7960 publications were eventually included, including 6503 “article” and 1457 “review” publications (Figure 1). The number of publications and citations was confirmed by quantitative analysis, which can objectively reflect the scientific research output and the current trend of mainstream research in a certain field. Our results indicated that the annual number of publications (Figure 2A) related to the TB vaccines and their citations (Figure 2B) showed an upward trend from 1995 to 2022, with a peak in 2021, with 484 publications and 26,324 citations, respectively.

### 3.2. Regional and Country Distribution of Studies Related to TB Vaccines

**Figure 3:**
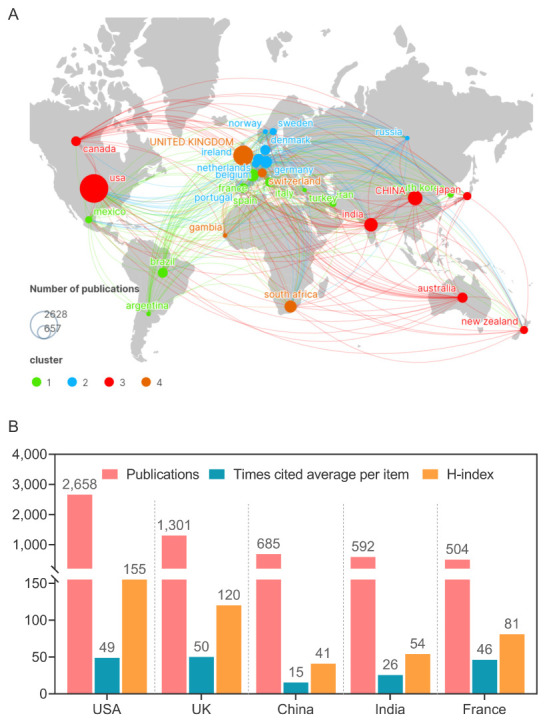
Regional and country distribution of studies related to TB vaccines. (**A**) Geographical distribution map based on the total number of publications of vaccines for TB in different countries. For the corresponding relationship between the number of national publications and the diameter of the ball, see the corner mark in the lower left corner. The national cooperation group is divided into four groups according to the ratio of the number of participating countries to the total countries count. The United States carries out the most cooperation from several national cooperation projects, followed by the United Kingdom and China. (**B**) Times cited average per item, publications, and H-index of the top 5 countries. The United States ranks first with 2658 publications, followed by the UK with 1301 publications. From the average number of citations per article, it can be found that there is little difference between the UK and the USA in this index. They shared the top leadership position. When we can roughly estimate the value of a country/discipline from the H-index, we find that the United States has the highest output value, with an H-index of 155. 5. UL: University of London; SSI: Statens Serum Institute; LSHTM: London School of Hygiene Tropical Medicine; UCT: University of Cape Town; UO: University of Oxford.

CiteSpace and Scimago Graphica were used to generate regional or country distribution maps to explore the contributions of different regions and countries in TB vaccine research. In this study, 30 out of 142 countries with more than 59 publications were divided into four clusters according to the ratio of the number of participating countries to the total countries counts (Figure 3A). Our results showed that: (1) Cluster 1 consisted of nine countries, including France, Brazil, Mexico, Italy, South Korea, Turkey, Iran, Argentina, and Spain; (2) Cluster 2 was made up of eight European countries (Norway, Sweden, Denmark, Ireland, Netherlands, Portugal, and Germany) and Russia; (3) Cluster 3 included the USA, China, India, Australia, Canada, New Zealand, and Japan; (4) Cluster 4 was dominated by the United Kingdom (UK), but also includes Switzerland, Gambia, and South Africa. The USA cooperated most among the multi-country cooperation projects, followed by the UK and China (Figure 3A). Furthermore, we also analyzed the number of publications, H-index, and times cited average per item among countries. The results indicated that five countries were leading the global trend in TB vaccine research, including the USA, UK, China, India, and France (Figure 3B): (1) Number of publications: the USA ranks first with 2658 articles, followed by the UK (1301), China (685), India (592), and France (504); (2) The average number of citations per article: UK (50) > USA (49) > France (46) > India (26) > China (15); (3) The H-index: USA (155) > UK (120) > France (81) > India (54) > China (41). These data suggest that the USA, the UK, and France are still the leaders in the field of TB vaccine research in the quantity and quality of publications. At the same time, India and China, as emerging countries, still have a certain gap compared with these developed countries. Especially in China, the number of publications has increased rapidly in recent years, but the quality of papers still needs further improvement.

### 3.3. Institutional Distribution in TB Vaccine Research

**Figure 4:**
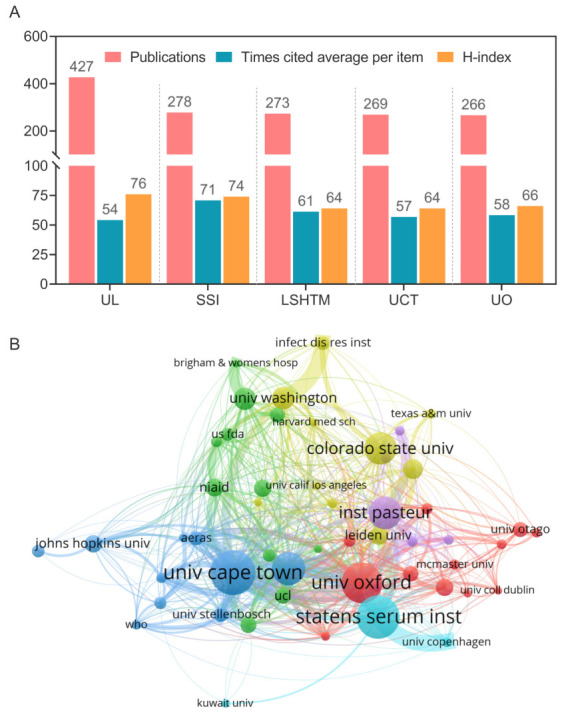
Map of the global distribution of institutions involved in TB vaccine research. (**A**) Times cited average per item, publications, and H-index of the top 5 institutions. UL: University of London; SSI: Statens Serum Institute; LSHTM: London School of Hygiene Tropical Medicine; UCT: University of Cape Town; UO: University of Oxford. (**B**) The inter-agency cooperation relationships from 1995 to 2022. We used the logarithmic likelihood ratio (LLR) to classify the research contents of each organization into six clusters, including cluster#0 in deep blue color, cluster#1 in light blue color, cluster#2 in green color, cluster#3 in yellow color, cluster#4 in red color, and cluster#5 in purple color.

Based on the regional and country distribution of studies related to TB vaccines, we further explored the institutional distribution in TB vaccine research (Figure 4A). Results showed that: (1) In terms of the number of publications, the University of London (UL) was far ahead with 427, followed by the Statens Serum Institute (SSI, *n* = 278), London School of Hygiene Tropical Medicine (LSHTM, *n* = 273), University of Cape Town (UCT, *n* = 269), and University of Oxford (UO, *n* = 266); (2) In terms of the number of citations per publication, the SSI occupied the first place (*n* = 71), followed by LSHTM (*n* = 61), UO (*n* = 58), UCT (*n* = 57), and UL (*n* = 54); (3) In terms of the H-index, UL (76) > SSI (74) > UO (66) > LSHTM (64) = UCT (64).

Moreover, the inter-agency cooperation relationships from 1995 to 2022 were analyzed by VOSviewer, and 52 of the 6382 institutions with more than 48 publications were classified into six clusters by the logarithmic likelihood ratio (LLR) (Figure 4B). Our results suggested that: (1) The most significant cluster #0 has 55 members, the most cited members in this cluster were UCT (*n* = 251), LSHTM (*n* = 185), and the University of Washington (UW, *n* = 118), and the most cited article of cluster #0 was a comparative study by Chelsea Carpenter et al., published in the journal *Tuberculosis* in 2015 [14]. (2) The second largest cluster #1 included 45 members, the most cited members in this cluster were SSI (*n* = 246), Institut Pasteur (IP, *n* = 172), and the University of Otago (*n* = 60), and the most cited article of this cluster was a study by Ann Williams et al., published in the journal *Tuberculosis* in 2005 [15]. (3) The third largest cluster #2 included 42 members, the most cited members in this cluster were the National Institute of Allergy and Infectious Diseases (NIAID, *n* = 91), University of Melbourne (UM, *n* = 81), and Harvard Medical School (*n* = 48), and the most cited article of this cluster was a review by Shabaana A Khader et al., published in the *Journal of Clinical Investigation* in 2019 [16]. The ranking of the top ten institutions’ centrality was shown in Table 1. The ten most influential institutions in the field of TB vaccine were UCT, LSHTM, UO, SSI, IP, Colorado State University (CSU), UM, NIAID, UCL, and Johns Hopkins University (JHU).

### 3.4. Funding Agencies Distribution in TB Vaccine Development

**Figure 5:**
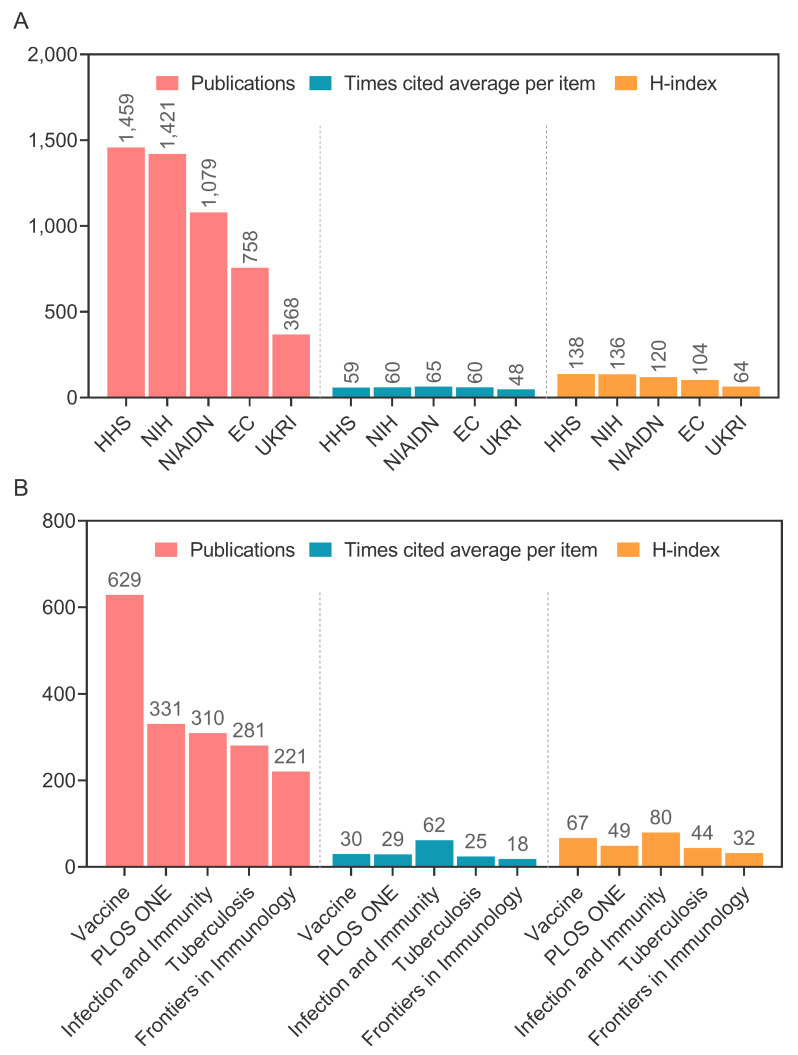
Top five funding agencies and their contributions to TB vaccine development. (**A**) The top five most active funding institutions in the field of TB vaccine. The output of the HHS and NIH was close to about 1400, NIAID got the highest number of cited times per article on average, and HHS got the highest H-index. (**B**) Times cited average per item, publications, and H-index of top 5 journals. Among the top five journals that published TB vaccine papers, *Frontiers in Immunology* received the highest IF (IF = 8.786). Regarding the number of journal publications, *Vaccine* ranked first with 629 articles. The average number of citations per article in *Infection and Immunity* was the highest (62.04). The H-index of *Infection and Immunity* was also the highest (80.5).

Funding is essential for vaccine development, which has been demonstrated in developing COVID-19 vaccines. Herein, our analysis illustrated the five most active funders of TB vaccine research (Figure 5). The results showed that the top five funding agencies in the field of TB vaccine research were the United States Department of Health and Human Services (HHS, *n* = 1459), NIH (*n* = 1421), NIAID (*n* = 1079), European Commission (EC, *n* = 758), and UK Research and Innovation (UKRI, *n* = 368) (Figure 5A). There was no significant difference in the number of citations among the publications funded by these five institutions, but there was a substantial difference in the H-index of the publications financed by these five institutions. These data suggest that five institutions have invested heavily in developing new TB vaccines, and the quality of the publications they have funded is high.

### 3.5. Analysis of Journals and Co-Cited Journals

Professional academic journals are essential carriers for the dissemination of academic papers. However, there are significant differences in the academic community’s reputation and recognition of different journals. Therefore, we systematically counted and analyzed the journals that published literature on TB vaccines, and compared the number of publications, the citations per publication, and the H-index of each journal (Figure 5B). The results showed that the top five journals with the largest number of publications on TB vaccine were *Vaccine* (*n* = 629), *PLoS ONE* (*n* = 331), *Infection and Immunity* (*n* = 310), *Tuberculosis* (*n* = 281), and *Frontiers in Immunology* (*n* = 221). In addition, *Infection and Immunology* had the most cited articles (*n* = 62) and the highest H-index (80) among these five journals.

**Figure 6:**
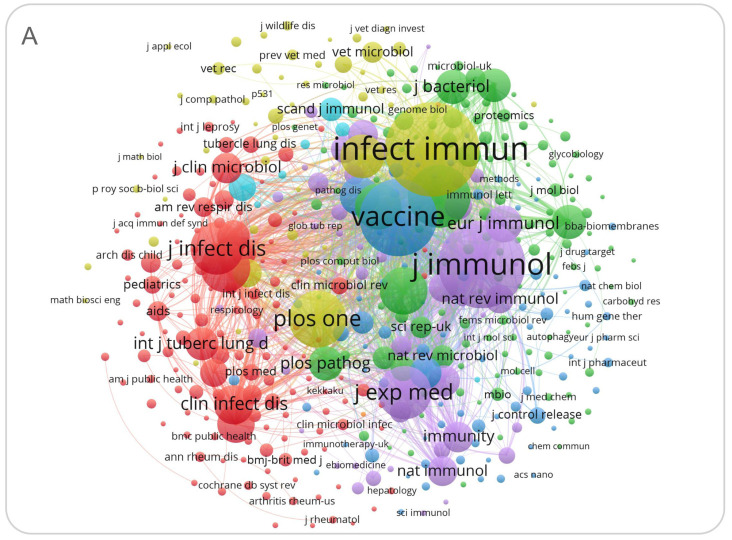

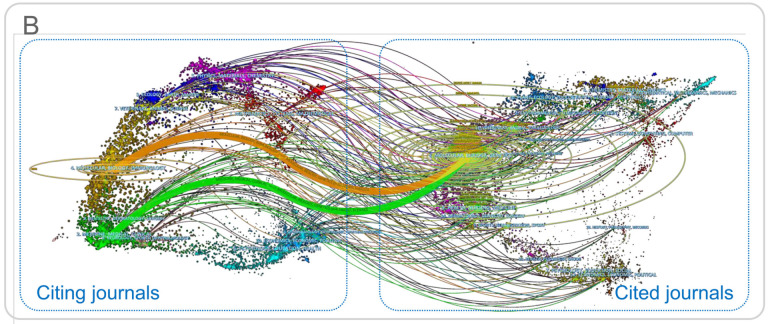
Co-citation analysis of journals and dual map of cited journals and cited journals. (**A**) Co-citation analysis of journals. Journals were divided into four clusters according to their preferred content. The co-citation between *Infection and Immunity* and *Journal of Immunology* was the most significant, showing that the contents of both journals are very similar. Furthermore, the three major journals that published articles on TB vaccines are *Infection and Immunity*, the *Journal of Immunology*, and *Vaccine*. (**B**) Dual map of journals in terms of citing journals and cited journals. (**B**) Two main pathways were observed, but both mainly cite the literature of the same discipline (molecular biology and genetics).

In addition, we also used the VOSviewer to conduct a co-citation analysis to observe the relationship between journals citing and being cited by each other. Our results revealed that 25 of the 24,002 relevant journals were cited more than 3000 times, and the top three highly cited journals were *Infection and Immunity*, *Journal of Immunology*, and *Vaccine* (Figure 6A). Then, a dual map overlay of the journals on targeted TB vaccines was constructed (Figure 6B). It was observed that: (1) There were two main pathways, such as the region of molecular biology and immunology and the region of medicine, pharmacy, and clinic. Interestingly, we found that both pathways mainly cited the literature of the same discipline (molecular biology and genetics); (2) The cited literature was also more or less related to other disciplines, mainly in the field of health, nursing, medicine, and veterinarians, animals, and parasites. This phenomenon indicated that clinical nursing work and the animals used in basic research had received significant attention. At the same time, the scope of influence of the cited literature (the number and size of circles in Figure 6B) also reflected that the cited literature mainly focused on molecular biology and genetics.

### 3.6. Distribution of Authors in Publications on TB Vaccines

**Figure 7:**
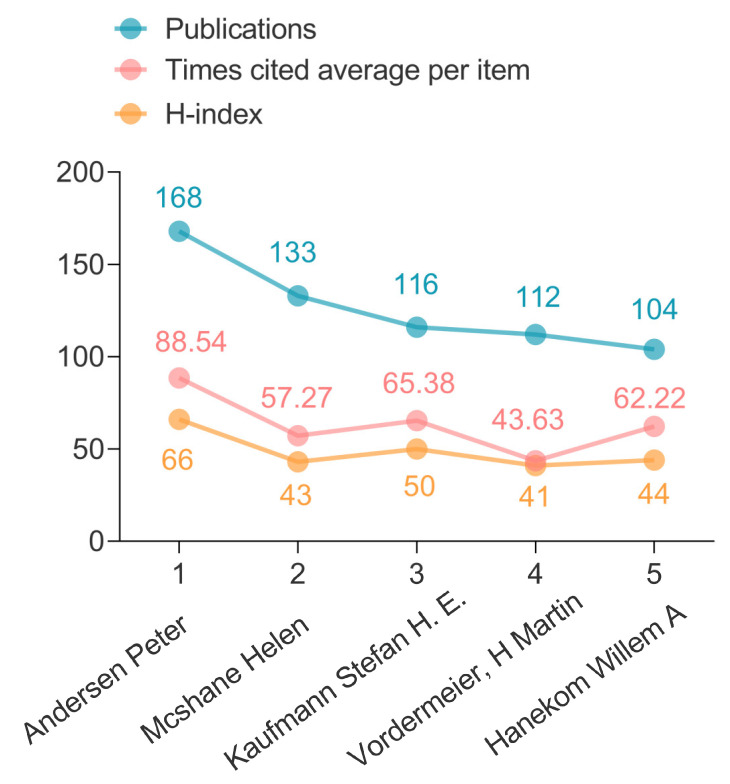
The top five most prolific authors in the field of TB vaccines over the past 27 years.

Figure 7 showed the top five prolific authors focused on TB vaccine research in the last 27 years, including Andersen Peter, Mcshane Helen, Kaufmann Stefan H. E., and Hanekom Willem A. Interestingly, Andersen Peter shared the highest number of publications (*n* = 168), the average number of citations per article (*n* = 88.54), and H-index (66) among all the authors.

### 3.7. Analysis of Reference Cluster

**Figure 8:**
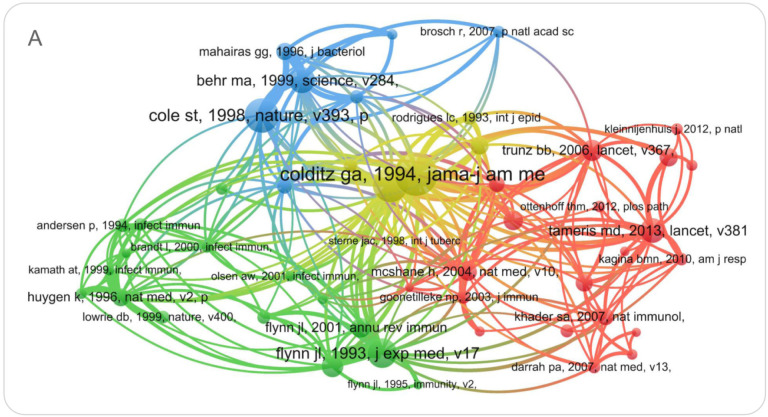

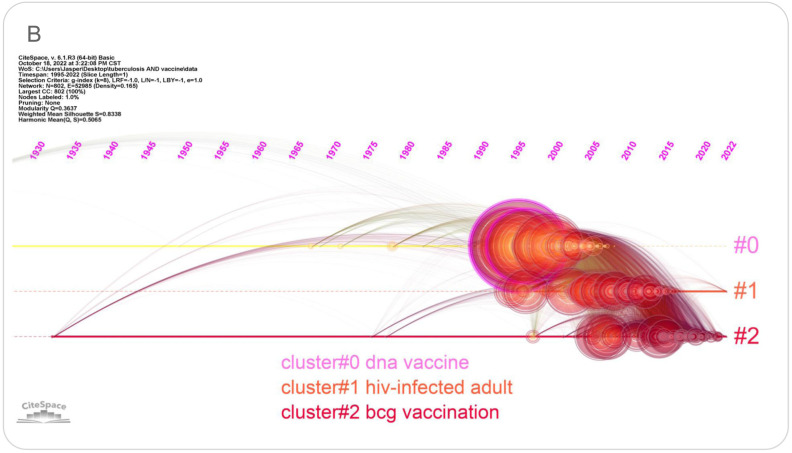
References cluster analysis and the results in the timeline view. (**A**) We use the logarithmic likelihood ratio (LLR) to classify the research contents of all references into three categories, including cluster#0 (yellow color) DNA vaccine, cluster#1 (green color) HIV-infected adult, and cluster#2 (red color) BCG vaccination. (**B**) The literature cited by the #0 cluster was ancient, with the farthest literature before 1920. The literature around 1995 significantly impacted the entire field of TB vaccines because the purple halo representing centrality was located in cluster #0. However, since 2005, the research popularity of the #0 cluster has declined. The research of cluster #1 began around 1995, and the popularity of related research has declined in the past decade. The study of cluster #2 started around 1930. The popularity of research is still very high since 2005. The references between the #0 and #2 clusters cited each other much more closely.

As shown in Figure 8A, the logarithmic likelihood ratio (LLR) was used to classify the research contents of all references into three categories, including cluster #0 (yellow color) DNA vaccine, cluster #1 (green color) HIV-infected adult, and cluster #2 (red color) BCG vaccination. (1) The largest cluster (#0) had 339 members, and three members received the most frequent citation, including “Efficacy of BCG Vaccine in the Prevention of Tuberculosis Meta-analysis of the Published Literature” published by Graham A. Colditz et al. in *JAMA* in 1994 (*n* = 804) [17], “Variation in protection by BCG: implications of and for heterologous immunity” published by P.E.M. Fine et al. in *Lancet* in 1995 (*n* = 643) [18], and “Deciphering the biology of *Mycobacterium tuberculosis (M. tuberculosis)* from the complete genome sequence” published by S. T. Cole et al. in *Nature* in 1998 (*n* = 623) [19]. (2) The second largest cluster (#1) had 236 members, and the most cited members in this cluster were “Immunology of Tuberculosis” published by JoAnne L. Flynn et al. in *Annu Rev Immunol* in 2001 (*n* = 355) [20], “The efficacy of bacillus Calmette-Guérin vaccination of newborns and infants in the prevention of tuberculosis: meta-analyses of the published literature” published by Colditz GA et al. in *Pediatrics* in 1995 (*n* = 321) [21], and “A multistage tuberculosis vaccine that confers efficient protection before and after exposure” published by Claus Aagaard et al. in *Nature Medicine* in 2011 (*n* = 315) [22]. (3) The third largest cluster (#2) had 227 members, and the top three members were “Safety and efficacy of MVA85A, a new tuberculosis vaccine, in infants previously vaccinated with BCG: a randomized, placebo-controlled phase 2b trial” published by Michele D Tameris et al. in *Lancet* in 2013 (*n* = 452) [23], “Effect of BCG vaccination on childhood tuberculous meningitis and miliary tuberculosis worldwide: a meta-analysis and assessment of cost-effectiveness” published by B Bourdin Trunz et al. in *Lancet* in 2006 (*n* = 384) [24], and “The success and failure of BCG—implications for a novel tuberculosis vaccine” published by Peter Andersen et al. in *Nature Review Microbiology* in 2011 (*n* = 363) [25]. We further analyzed references on the timeline based on CiteSpace. As shown in Figure 8B, in terms of the distribution of the entire timeline, the literature can be divided into three clusters: #0 DNA vaccine, #1 HIV-infected adult, and #2 BCG vaccination.

### 3.8. Analysis of Being Cited Status of TB Vaccine-Related Studies

Table 2 summarized the top 10 most frequently cited original articles on TB vaccines. “Randomized, controlled trials, observational studies, and the hierarchy of research designs,” published by Concato, J et al. in the *New England Journal of Medicine* in 2000, received the most frequent citation (*n* = 2305) [26]. This comparative study used published meta-analysis data to identify and explore randomized clinical trials and observational studies on the same clinical topic, comparing results from original reports according to the type of study design. They found that the results of well-designed observational studies did not systematically overestimate the magnitude of the treatment effect when compared with randomized controlled trials on the same topic. The remaining nine highly cited articles focused on immune response [20,27,28,29], BCG [18,30], DNA vaccine [31], methodology [32], and treatment of rheumatoid arthritis [33], respectively.

### 3.9. Burst References Analysis

**Figure 9:**
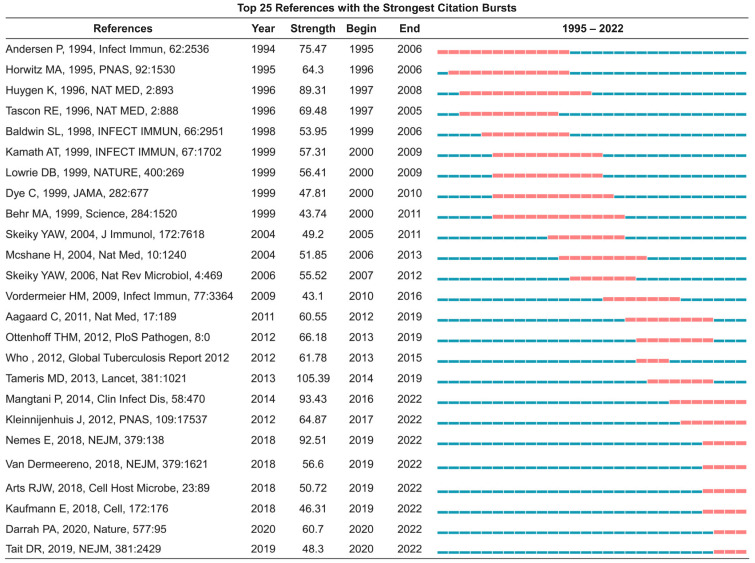
**Top 25 most cited references.** The literature that is still in the period of citation explosion is as follows: [36,37,38,39,40,42,43,44]. This future research hotspot is hidden in these documents.

Burst detection is an algorithm developed by Kleinberg [34]. Kleinberg’s burst detection algorithm is considered a powerful tool to calculate and identify the frontier or emerging trend of research on the timeline. We use this algorithm to determine the key references and keywords related to the TB vaccines. As a result, the top 25 references with the most powerful citation bursts were identified via CiteSpace (Figure 9). Among the above 25 works of literature, the top one was a research article published by P Andersen in *Infection and Immunity* in 1994 [35]. This study evaluated the protective efficacy of a short-term culture filtrate (ST-CF) vaccine containing proteins secreted from actively replicating mycobacterial growth and found that the protective effect of the ST-CF vaccine was comparable to that of the standard BCG vaccine [35].

Interestingly, we observed that eight pieces of literature were still in the period of citation explosion, including four publications focusing on the BCG vaccine [36,37,38,39], one article evaluating the protective efficacy of the H4:IC31 vaccine [40], one article exploring the association between genetic polymorphisms and TB susceptibility [41], and two clinical trials validating the protective efficacy of M72/AS01E [42,43]. The M72/AS01E vaccine was once considered the most promising new vaccine to prevent and control the spread of TB. Unfortunately, the final analysis of a randomized controlled clinical trial published in the *New England Journal of Medicine* in 2019 showed that its efficacy at month 36 was only 49.7% [43], which was lower than the 50% protection efficiency stipulated by the WHO.

### 3.10. Trend Topics and Thematic Maps Analysis

**Figure 10:**
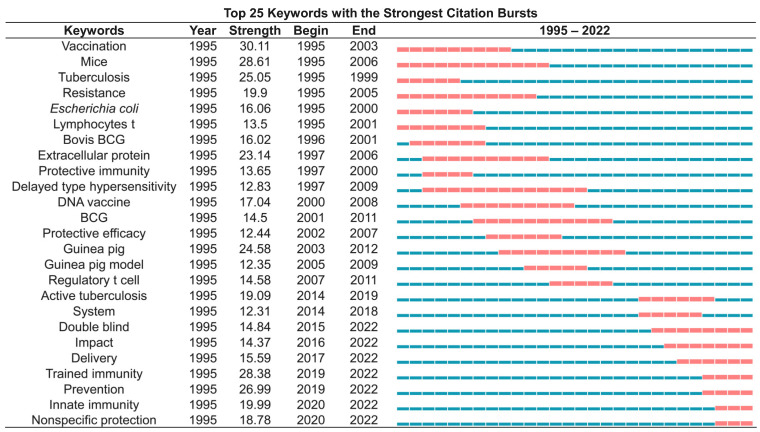
Top 25 most cited keywords. The year with a particular keyword still in citation bursts was shown in red, and others were shown in blue.

Although the above results comprehensively reviewed TB vaccine research history and the current situation, they could not predict the future development trend. In this study, burst keywords and thematic maps were used to analyze and predict future development trends and hotspots in the TB vaccine research field. The burst keywords in the publications on TB vaccines were detected by CiteSpace. The results revealed that 25 burst keywords with the highest citation strength were identified from 1995 to 2022 (Figure 10). Of these burst keywords, “vaccination” had the highest strength (30.11) from 1995–2003. At present, seven keywords are still in the period of citation explosion, including “double-blind”, “impact”, “delivery”, “trained immunity”, “prevention”, “innate immunity”, and “nonspecific protection”.

**Figure 11:**
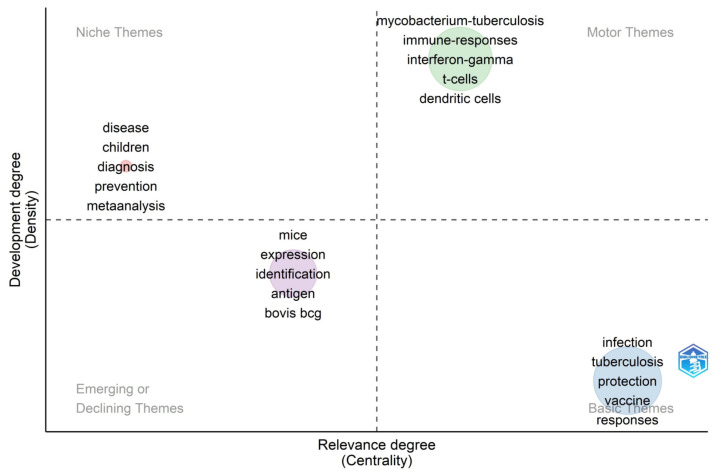
Thematic Map. In the thematic map, the horizontal axis represents the centrality, the vertical axis represents the density, and four quadrants were drawn accordingly. The words in the first quadrant (motor themes) indicate that they are essential to TB vaccine research and are well developed, representing the future development trend. The words in the second quadrant (Niche themes) suggest that although they have been well developed, they are not crucial for the current research field. The words in the third quadrant (emerging or themes) indicate that they may be new research fields that have just emerged or past research fields that are about to disappear. Finally, the words in the fourth quadrant (basic themes) indicate that they are essential to TB vaccine research but are not well developed.

Thematic maps are often used to predict future research trends and hot areas in a certain field [45]. Our results showed that: (1) The most promising research directions and hotspots in the field of TB vaccine research in the future will involve keywords including “*M. tuberculosis*”, “immune responses”, “interferon gamma”, “T cells”, and “dendritic cells” (Figure 11, Motor-Themes); (2) Five keywords have been used frequently in the past, but will be used less frequently in the future, including “ disease “, “children”, “diagnosis”, “prevention”, and “meta-analysis” (Figure 11, Niche-Themes); (3) Five keywords are in decline, including “mice,” “expression,” “identification,” “antigen,” and “bovis BCG” (Figure 11, Emerging or Declining Themes); (4) Studies involving the following five keywords have not been paid attention to although they are crucial to TB vaccine research, including “infection”, “tuberculosis”, “protection”, “vaccine”, and “responses” (Figure 11, Basic-Themes).

By analyzing these keywords, it was found that the early studies paid more attention to evaluating TB vaccine protection in animal models. In contrast, the latest studies turned to explore the interaction mechanism between the TB vaccine and the host, such as ”trained immunity”, “innate immunity”, “immune responses”, “interferon gamma”, “T cells”, and “dendritic cells”.

## 4. Discussion

This study first employed bibliometrics to summarize the status and predict future trends in the field of TB vaccine research. The rapid development of information technology and artificial intelligence technology has given wings to the leap from traditional literature descriptive analysis to digital and standardized bibliometrics [46,47]. Unlike the conventional descriptive review, systematic review, and meta-analysis, bibliometric analysis can process and extract massive literature data to panoramic research status and predict future trends [48]. Unfortunately, no literature provides a bibliometric analysis of the field of TB vaccine research. Therefore, we conducted a bibliometric study based on 7960 articles published between 1995 and 2022 by using VOSviewer, CiteSpace, Scimago Graphica, and Bibliometrix to provide new insights into TB vaccine development.

From 1995 to 2022, the total number of publications on TB vaccines was 7960, including 6503 articles and 1457 reviews. Our results revealed that the annual number of publications on TB vaccines and their citations showed an upward trend from 1995 to 2022, with a peak in 2021, with 484 publications and 26,324 citations, respectively (Figure 2). Interestingly, the annual number of publications and citations rose steadily from 1995 to 2011, but both declined in 2012 (Figure 2). This may be closely related to the DNA vaccine research boom in the late 20th century (Figure 8B). We speculated that the rise of a new research field would come at the expense of the decline of other research fields. Specifically, the new research field will attract many researchers to abandon their previous research directions, eventually leading to a temporary reduction in the number of publications and citations. We found that the number of publications on the TB vaccine decreased briefly in 2018 and then increased rapidly, which may be related to several events: (1) On 5 October 2018, The European Medicines Agency’s (EMA) Committee for Medicinal Products for Human Use (CHMP) has recommended granting marketing authorization for the gene therapy Luxturna (voretigene neparvovec [49,50,51]), for the treatment of adults and children suffering from inherited retinal dystrophy caused by *RPE65* gene mutations, a rare genetic disorder which causes vision loss and usually leads to blindness [52]; (2) CRISPR: The gene-editing tool revolutionizing biomedical research [53,54,55]: a new tool could be the key to treating genetic diseases and may be the most consequential discovery in biomedicine this century (https://www.cbsnews.com/video/crispr-the-gene-editing-tool-revolutionizing-biomedical-research/, accessed on 10 January 2023); (3) Cynthia E Dunbar, etc., published an article titled “Gene therapy comes of age” on Science [56]. This study summarized the gene therapies that have recently been approved to use in hereditary immune diseases, hemophilia, eye, and neurodegenerative diseases, and lymphatic cancer, which might have attracted researchers’ attention to exploring gene therapies. Therefore, the number of publications and citations decreased sharply in 2018–2019.

People easily ignore the above scientific events, but the development of bibliometrics provides a tool for people to sort out the impact of these complex scientific events on the development of science based on the indicators such as the number of publications, the average number of citations, H-index, and centrality. Based on the above three fundamental indicators, the bibliometric data of the top five journals, countries, institutions, and authors were analyzed (Table S1). Academic journals are the best stage for scientists to exchange research results and disseminate scientific knowledge [57]. The number of journals in a particular field, especially the number of international journals, is related to the country’s status in this field to a certain extent [58]. For example, the United States and the United Kingdom, respectively, own two top journals in the field of TB vaccines, including *PloS ONE* (USA), *Infection and Immunity* (USA), *Vaccine* (UK), and *Tuberculosis* (UK). As expected, the USA and the UK are the greatest beneficiaries of these four most influential journals in vaccinology (Table S1).

Furthermore, we also found that in terms of the number of publications (Figure 3), the USA, UK, and France were ranked in the top five, which might be closely related to their long-term, in-depth research on TB vaccines, advanced experimental equipment, and sufficient personnel and financial investment [59,60,61,62,63,64,65]. In contrast, among developing countries, only China and India made it into the top five, mainly owing to the realities of their high TB burden (Table S1). According to the report published by WHO in 2022, India is a significant contributor to the global incidence of TB, followed by China [1]. Interestingly, we found that although China and India ranked third and fifth in the world in terms of the number of publications, respectively, they fell down the rankings when measured by quality indicators (average citation frequency and H-index) for scientific contribution (Figure 3). These data indicated that the developed countries, led by the USA and the UK, are still leading the way in TB vaccine research, whereas the developing countries, led by China and India, are catching up and gradually increasing their impact on TB vaccine research.

The national scientific and technological outputs in the TB vaccine research field are composed of various institutions’ outcomes. Our study found that the most active institutions globally in TB research were the University of London, London School of Hygiene & Tropical Medicine, the University of Oxford in the UK, Statens Serum Institut in Denmark, and the University of Cape Town in South Africa (Figure 4). In addition, our data also showed that Mcshane Helen and Hanekom, Willem A, two researchers with a high number of publications and a high H-index, were the leaders in TB vaccine research. Both researchers were affiliated with the University of Cape Town, the University of Oxford, and the University of London.

The number of publications reflects the publication contributions of a country, institution, or individual but not the impact of those contributions. To overcome this drawback, the average citations per article [66,67] and H-index [68,69,70,71,72] were introduced to the quality evaluation. In this study, countries were ranked based on the contribution percentage, and we found that the top five countries with outstanding contributions to TB vaccine research were the USA, the UK, France, Germany, and Denmark (Figure S1). All of them are developed countries, but the output in developing countries or regions, including South Africa, China, and India, accounted for a substantial share as well. Developing countries did not perform well in the value evaluation system, but this did not fully mean that the output value of developing countries was necessarily poor [73,74,75,76,77,78]. To further analyze the differences in research content between developing and developed countries, we took the USA and the UK as representatives of typically developed countries and China and India as representatives of typically developing countries. Cluster analysis was performed on the specific research content. Using Bibliomatrix’s thematic evolution analysis module, we obtained an analysis of the overall research direction of TB vaccines in these four countries (Figures S2–S9). At the same time, combined with the performance of institutions and individuals in these four countries in the world, we believe that one of the major reasons for the low value of scientific research outputs in China (Figures S4 and S8) and India (Figures S5 and S9) was that their institutions and individuals were all-round development without a powerful center to lead just like the light tower. Meanwhile, the level of input in some key areas was not as deep as that of developed countries such as the USA (Figures S2 and S6) and the UK (Figures S3 and S7). So the fact that developed countries are far ahead in the global ranking of the scientific research value of institutions and individuals makes sense [79].

Finally, we have analyzed the interesting research differences among the most important institutions in the TB vaccine research field by using CiteSpace (Figures S10–S20). The University of London (Figure S10) has recently been working on the TB vaccine secreted protein, how to use the TB vaccine to overcome tuberculosis, the relationship between the TB vaccine and *M. tuberculosis* survival, the agenda of tuberculosis vaccine development, the latest progress (intracellular immunity, BCG vaccine, school-age children, cytokines, mortality, etc.), vaccine response, TB vaccine, and inert biological products. Statens Serum Institut (Figure S11) has recently been working on a healthy neonatal tuberculosis vaccine, the development of a vaccine based on a mouse TB infection model, and a new adjuvant formula for the TB vaccine. London School of Hygiene & Tropical Medicine (Figure S12) focuses on reinfection after TB vaccine injection, registered other bacilli, BCG vaccination response, tuberculous meningitis, vaccine response, and multiple infection complex. The University of Cape Town (Figure S13) focuses on vaccine development, BCG vaccination, infants vertically infected with HIV, young children, vaccine efficacy, social behavior, adolescents, and time trends of infection. The University of Oxford (Figure S14) has been carried out in many directions, including *M. tuberculosis* antigen 85a, candidate vaccine MVA85A, LTBI (latent TB infection), factors affecting prognosis, epidemiology of infectious diseases, co-infection of COVID-19, secondary molecules, proteins, and cytokines in vaccine development, rope coefficient, recombinant BCG vaccine, and influenza virus 17D, CD8^+^ T cells in the respiratory system. It is obvious that the top TB vaccine research institutions are focused on: (1) vaccine efficacy, vaccination, and post-vaccination response; (2) targeted vaccines for infection in young children and adolescents, trials of existing candidate vaccines; and (3) cellular and molecular biological mechanisms of vaccines and diseases. Drawing lessons from top institutions’ research hotspots may help us carry out our new work and determine the new direction, which is the most fundamental significance of the bibliometric analysis of the TB vaccine. Furthermore, the analysis results of the other six institutions are presented in Figures S15–S20.

This study has some limitations: (1) Only the SCI-E database was used for literature retrieving, resulting in missing some publications included in other databases; (2) Literature in English rather than other language were included in this study, which may result in a bias on publications selection; (3) The time span was set between 1995–2022, and the literature published before 1995 was not considered; (4) Some high TB burden countries with few publications on TB vaccines cannot be analyzed by using bibliometrics, such as Pakistan, Indonesia, Philippines, Bangladesh, Lesotho, Myanmar, Mongolia, and Vietnam.

## 5. Conclusions

The number of publications on TB vaccines has increased over the past two decades. Meanwhile, the amount of knowledge passed down by researchers from generation to generation is enormous. How to extract the essence and discard the dregs is where bibliometrics comes into its own. Developed countries play a significant role in TB vaccine research, and developing countries are rapidly catching up. Future research trends and hotspots should focus on understanding the interaction mechanism between the TB vaccine and the host, and conducting more extensive clinical trials.

## Figures and Tables

**Table 1:** The ranking of the top ten institutions of centrality.

Publications	Centrality ^a^	Institution	Year	Half-life ^b^	Cluster ID ^c^
251	0.13	UCT	2005	9.5	0
185	0.13	LSHTM	1998	18.5	0
236	0.12	UO	1998	15.5	4
246	0.12	SSI	1998	14.5	1
172	0.1	IP	1998	7.5	1
184	0.09	CSU	1998	12.5	3
81	0.06	UM	2009	7.5	2
91	0.06	NIAID	1998	16.5	2
75	0.05	UCL	1998	16.5	5
84	0.05	JHU	1999	13.5	0

^a^. Centrality represents the influence of the analysis object on the whole analysis result. A total of 6382 research institutions were included in the overall network analysis results. The average centrality of all institutions was 0.02. The higher the centrality, the greater the influence on the whole field of research. ^b^. Half-life means the amount of time that an organization produces articles from when the number of citations increases until the number of citations gradually approaches the baseline value. ^c^. Cluster ID indicates the serial number of the cluster to which the organization belongs in cluster analysis. UCT: University of Cape Town; LSHTM: The London School of Hygiene & Tropical Medicine; UO: University of Oxford; SSI: Statens Serum Institute; IP: Institute Pasteur; CSU: Colorado State University; UM: University of Melbourne; NIAID: National Institute of Allergy and Infectious Diseases; UCL: London’s Global University; JHU: Johns Hopkins University.

**Table 2:** The top 10 most cited original articles related to the vaccines for TB.

Rank	Reference	Publish Time	2018	2019	2020	2021	2022	Average per Year	Total
1	[26]	Jun 2000	97	102	102	99	60	100.22	2305
2	[20]	2001	43	37	40	45	32	74.27	1634
3	[27]	Aug 2010	127	104	99	112	56	93.92	1221
4	[30]	May 1999	23	25	18	22	14	49.5	1188
5	[33]	Jan 2016	280	273	180	166	87	172.43	1207
6	[28]	Apr 2008	68	55	55	64	49	75.07	1126
7	[32]	Feb 2002	65	68	60	65	40	53.57	1125
8	[29]	Jul 2007	52	55	49	51	16	64.31	1029
9	[18]	Nov 1995	30	32	39	46	16	35.93	1006
10	[31]	2000	21	12	14	11	5	41.39	952
Accumulation	10 Publications		806	763	656	681	375	760.61	12,793
Total	7960 Publications		18,944	20,042	23,584	26,323	17,733	10,302.79	288,478
Ratio	0.125%		4.25%	3.81%	2.78%	2.59%	2.11%	7.38%	4.43%

## Data Availability

All data generated or analyzed during this study are included in the public database.

## Supplementary Materials

The following supporting information can be downloaded at: https://www.mdpi.com/article/10.3390/jpm13030408/s1, Figure S1. Pie chart of contribution percentage of nine countries with advantages to tuberculosis vaccine research. Figure S2. (A). Cluster analysis of research content of the USA; (B). Cluster analysis in the timeline view. Figure S3. (A). Cluster analysis of research content of the UK; (B). Cluster analysis in the timeline view. Figure S4. (A). Cluster analysis of research content of China; (B). Cluster analysis in the timeline view. Figure S5. (A). Cluster analysis of research content of India; (B). Cluster analysis in the timeline view. Figure S6. Thematic evolution map of tuberculosis vaccine research in the USA. Figure S7. Thematic evolution map of tuberculosis vaccine research in the UK. Figure S8. Thematic evolution map of tuberculosis vaccine research in China. Figure S9. Thematic evolution map of tuberculosis vaccine research in India. Figure S10. Progress map of the research topic of UL (from the UK, ranked global No.1) on TB vaccines in the past ten years. Figure S11. Progress map of the research topic of SSI (from Denmark, ranked global No.2) on TB vaccines in the past ten years. Figure S12. Progress map of the research topic of LSHTM (from the UK, ranked global No.3) on TB vaccines in the past ten years. Figure S13. Progress map of the research topic of UCT (from South Africa, ranked global No.4) on TB vaccines in the past ten years. Figure S14. Progress map of the research topic of UO (from the UK, ranked global No.5) on TB vaccines in the past ten years. Figure S15. Progress map of the research topic of Fudan University (from China) on TB vaccines in the past ten years. Figure S16. Progress map of the research topic of APHA (from the UK) on TB vaccines in the past ten years. Figure S17. Progress map of the research topic of the University of Harvard (from USA) on TB vaccines in the past ten years. Figure S18. Progress map of the research topic of ICMR (from India) on TB vaccines in the past ten years. Figure S19. Progress map of the research topic of Max Plank Society (from Germany) on TB vaccines in the past ten years. Figure S20. Progress map of the research topic of UDICE (from French) on TB vaccines in the past ten years. Table S1. The relevant grade distribution map is concerned with the exciting program with any one advantage in the top five journals, the top five countries, the top five institutions, and the top five individuals in the research area of tuberculosis vaccines.
