# Supplementary material for: A Summary on Tuberculosis Vaccine Development—Where to Go?
Source: J Pers Med. 2023 Feb 24;13(3):408. doi: 10.3390/jpm13030408 (PMC10054751; doi:10.3390/jpm13030408)
Supplement: Supplementary file 1 [file jpm-13-00408-s001.zip › Supplementary material/Table S1.docx]

| **Tabel S1: Discussion** | | | | |
| --- | --- | --- | --- | --- |
| **Journal** | **Country** | **Institution** | **Author** | **Address** |
| Plos One(USA) | USA(2660,49.06,155) | Harvard University(258,64.18,64) | Fortune, Sarah M.(17,65.94,13） | 2022-2022 Dalhousie University 2022-2022 Harvard 2022-2022 Harvard Medical School 2022-2022 University of Pittsburgh 2014-2022 Harvard T.H. Chan School of Public Health 2013-2022 Ragon Institute 2013-2022 Broad Institute 2012-2022 University of British Columbia 2012-2022 Massachusetts Institute of Technology (MIT) 2004-2022 Harvard University 2020-2021 Fisheries & Oceans Canada 2020-2020 University of Johannesburg 2017-2020 Woods Hole Oceanographic Institution 2017-2017 Clin Practice Guideline Self Harm Working Comm 2017-2017 University of Auckland 2016-2016 Bell Labs 2016-2016 Google Incorporated 2015-2015 Massachusetts General Hospital 2000-2000 Brigham & Women's Hospital |
| Infection and Immunity(USA) |  |  |  |  |
| Vaccine(UK) | England(1301,50.1,120) | University of London（421,54.62,75) | Dockrell, Hazel M.(49,43.92,24) | 1989-2022 London School of Hygiene & Tropical Medicine 2020-2020 MRC Uganda Virus Res Inst AIDS 2016-2016 University of London Royal Veterinary College 2015-2015 Aeras 2015-2015 MRC 2009-2009 Anandaban Hosp 2008-2008 Dept Infect & Trop Dis 1980-1985 UCL Medical School 1983-1983 University of London |
|  |  | London School of Hygiene & Tropical Medicine(267,62.09,63) |  |  |
|  |  | University of Oxford(265,58.3,65) | Mcshane, Helen(129,57.79,43) | 2001-2022 University of Oxford 2021-2021 NIHR Oxford Biomed Res Ctr BRC 2011-2021 Oxford University Hospitals NHS Foundation Trust 2020-2020 Jenner Inst Oxford 2009-2017 Jenner Institute 2015-2016 Aeras 2014-2014 Oxford Univ Hosp 2013-2014 University of Cape Town 2008-2008 Aeras Global TB Vaccine Fdn 2005-2005 Radcliffe Infirmary |
| Tuberculosis(UK) |  | Animal and Plant Health Agency, APHA(6,7.5,3) | Vordermeier, H Martin | 2015-2018 Animal & Plant Health Agency UK 2017-2017 APHA 2015-2015 APHA Weybridge 2015-2015 UK Vet Labs Agcy 1998-2015 Veterinary Laboratories Agency 2014-2014 AHVLA Weybridge 2012-2014 Anim Hlth & Vet Labs Agcy 2012-2014 AHVLA 2013-2013 Agcy Weybridge 2012-2012 Anim Hlth & Vet Labs Agcy AHVLA Weybridge 2004-2012 Vet Lab Agcy 2011-2011 Anim Hlth Vet Lab Agcy Weybridge 2011-2011 Vet Labs Agcy VLA Weybridge 2002-2011 TB Res Grp 2002-2010 VLA Weybridge 2009-2009 UK Vet Lab Agcy 1999-1999 Vet Lab Agcy Weybridge 1995-1997 Imperial College London 1995-1995 MRC 1987-1987 Eberhard Karls University of Tubingen |
|  | China(684,15.45,41) | Fudan University(74,18.95,21) | Xu, Ying(22,17.64,13) | Fudan University Zhongshan Xuhui Hospital |
|  | India(593,25.67,54) | INDIAN COUNCIL OF MEDICAL RESEARCH ICMR(114,42.16,31) | Gupta, Umesh D. (17,25.47,13) | ICMR - National JALMA Institute for Leprosy & Other Mycobacterial Diseases, Agra AGRA, UTTAR PRADESH, INDIA |
|  | France(503,46.47,81) | UDICE FRENCH RESEARCH UNIVERSITIES(179,49.34,46) | Casanova, Jean-Laurent(23,134.7,18) | 2017-2017 Instituto Universitario de Lisboa 2017-2017 Rockefeller Branch 2013-2017 Howard Hughes Medical Institute 2009-2017 Rockefeller University 2007-2017 Institut National de la Sante et de la Recherche Medicale (Inserm) 2001-2017 Universite Paris Cite 2000-2017 UDICE-French Research Universities 1995-2017 Hopital Universitaire Necker-Enfants Malades - APHP 2016-2016 KU Leuven 2016-2016 University Hospital Leuven 2014-2014 CHU Grenoble Alpes 2008-2014 King Saud University 2007-2011 Shanghai Jiao Tong University 2008-2008 Pediat Hematol Immunol Unit 2004-2004 NIH National Institute of Allergy & Infectious Diseases (NIAID) 2001-2001 Fac Med Necker Enfants Malades 1992-1993 Ludwig Institute for Cancer Research 1991-1991 University of Lausanne 1990-1990 Institut Pasteur Paris |
|  | The Kingdom of Denmark(338,66.52,79) | Statens Serum Institut(278,70.9,74) | Andersen Peter(164,86.73,62) | 1991-2020 Statens Serum Institut 2013-2019 University of Copenhagen 2016-2016 Boston Children's Hospital 2009-2009 Dept Infect Dis Immunol |
|  | South Africa(472, 47.84,77) | University of Cape Town(269,57,64) | Mcshane, Helen(32,60.44,17) | 2001-2022 University of Oxford 2021-2021 NIHR Oxford Biomed Res Ctr BRC 2011-2021 Oxford University Hospitals NHS Foundation Trust 2020-2020 Jenner Inst Oxford 2009-2017 Jenner Institute 2015-2016 Aeras 2014-2014 Oxford Univ Hosp 2013-2014 University of Cape Town 2008-2008 Aeras Global TB Vaccine Fdn 2005-2005 Radcliffe Infirmary |
|  |  |  | Hanekom, Willem A.(102,63.24,44) | 2021-2022 University College London 2020-2022 Africa Health Research Institute 1997-2022 University of Cape Town 2014-2019 Bill & Melinda Gates Foundation 2008-2013 Sch Child & Adolescent Hlth 2011-2011 SATVI 2009-2009 S African TB Vaccine Initiat 2008-2008 McGill University 2003-2004 University of Miami 1999-2001 Rockefeller University 1995-1995 Northwestern University |
|  | Germany(456,53.92,81) | MAX PLANCK SOCIETY(132,61.32,50) | Kaufmann, Stefan H. E.(113,65.97,50) | 2022-2022 Max Planck Inst Multidisciplinary Sci 2019-2022 Texas A&M University System 1982-2022 Max Planck Society 2020-2020 Texas A&M Vet Med 2019-2019 Aarhus University 2012-2019 Charite Universitatsmedizin Berlin 2015-2015 Aeras 2013-2014 University of Melbourne 2004-2005 MPI Infect Biol 1988-1998 Ulm University 1988-1988 ABT MED MIKROBIOL IMMUNOL 1987-1987 ABT MED MIKROBIOL & IMMUNOL 1979-1984 Free University of Berlin 1982-1982 BASEL INST IMMUNOL |
| Frontiers in Immunology(Switzerland) | Switzerland(269,67.36,70) | WORLD HEALTH ORGANIZATION(72,103.47,39) | Dye, Christopher(11,260,10) | 1982-2021 University of Oxford 1998-2019 World Health Organization 2018-2018 Univ Southern Oregon 2016-2016 University of Bern 1987-1996 London School of Hygiene & Tropical Medicine 1994-1994 University of Cambridge |
