# Supplementary figures and images for: A Summary on Tuberculosis Vaccine Development—Where to Go?
Source: J Pers Med. 2023 Feb 24;13(3):408. doi: 10.3390/jpm13030408 (PMC10054751; doi:10.3390/jpm13030408)

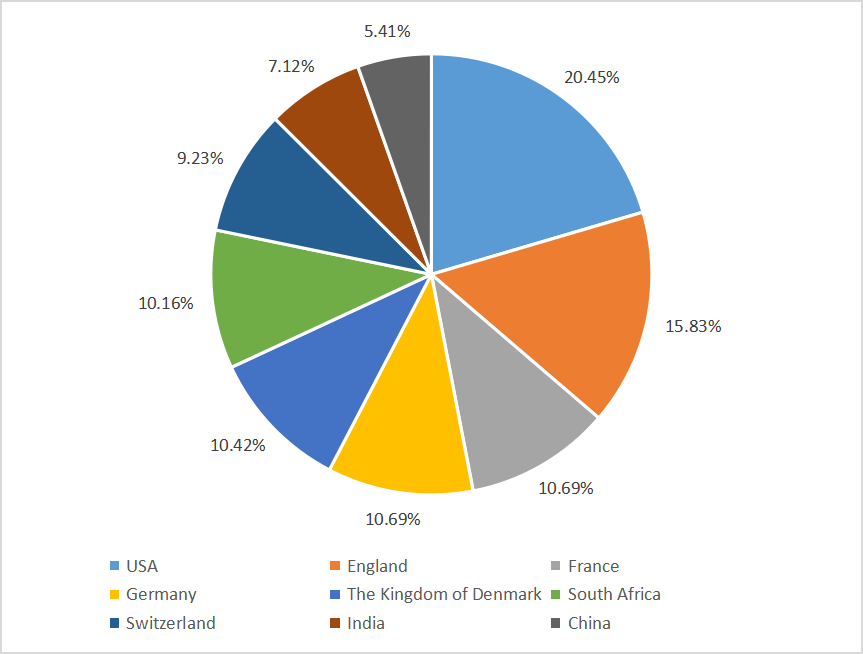

Supplement: Supplementary file 1 [file jpm-13-00408-s001.zip › Supplementary material/Figrue S1.png]

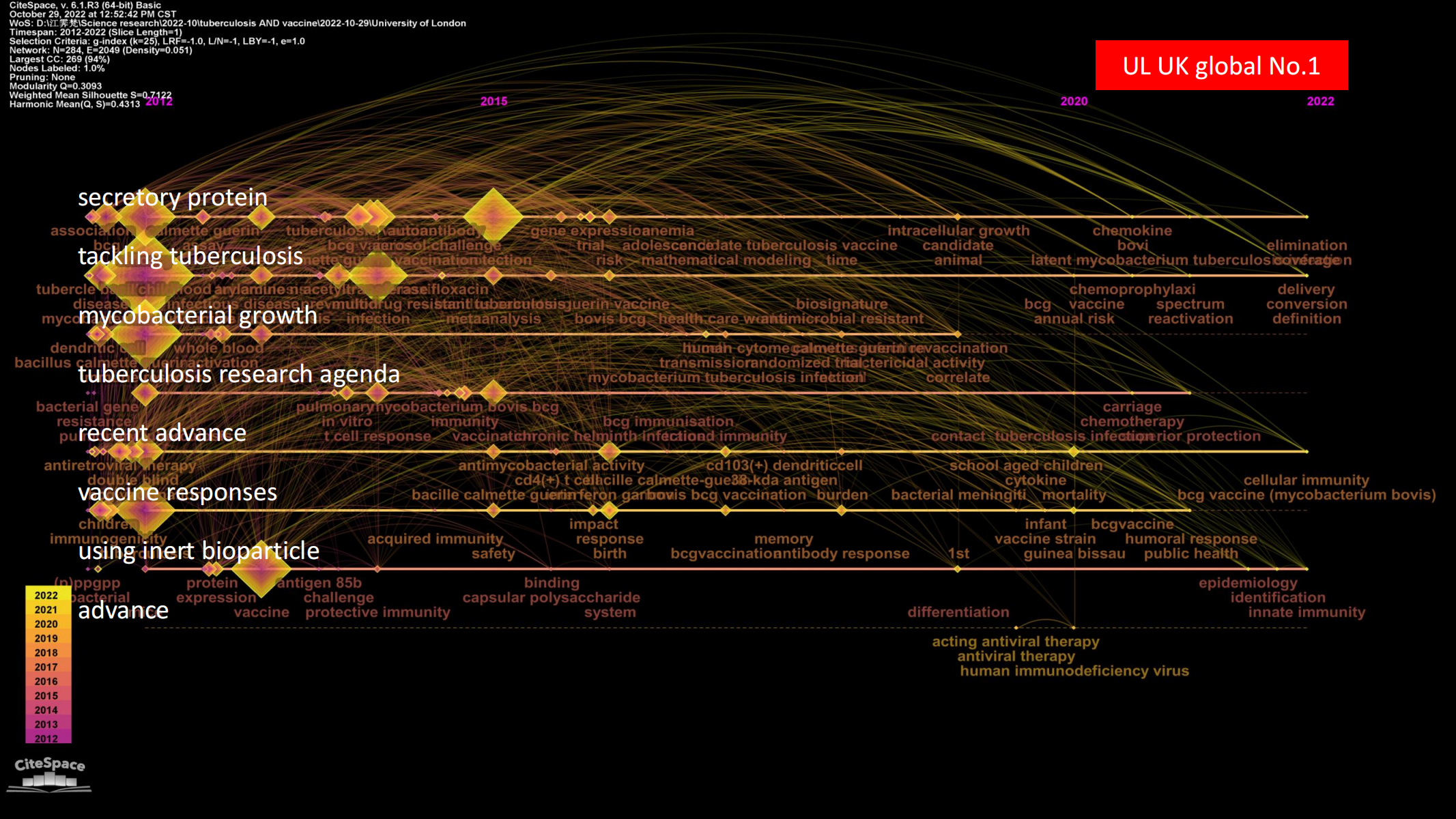

Supplement: Supplementary file 1 [file jpm-13-00408-s001.zip › Supplementary material/Figure S10.png]

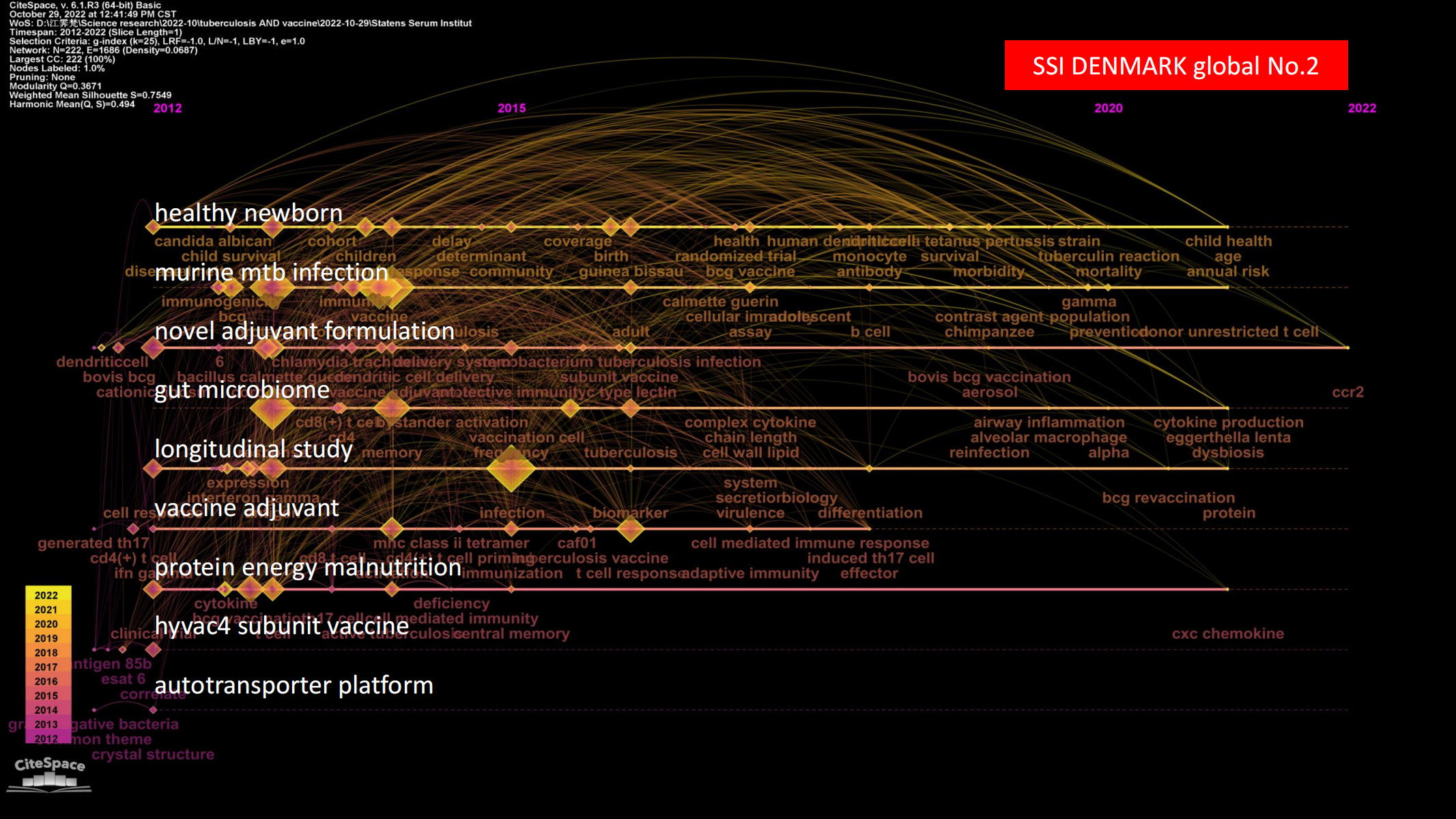

Supplement: Supplementary file 1 [file jpm-13-00408-s001.zip › Supplementary material/Figure S11.png]

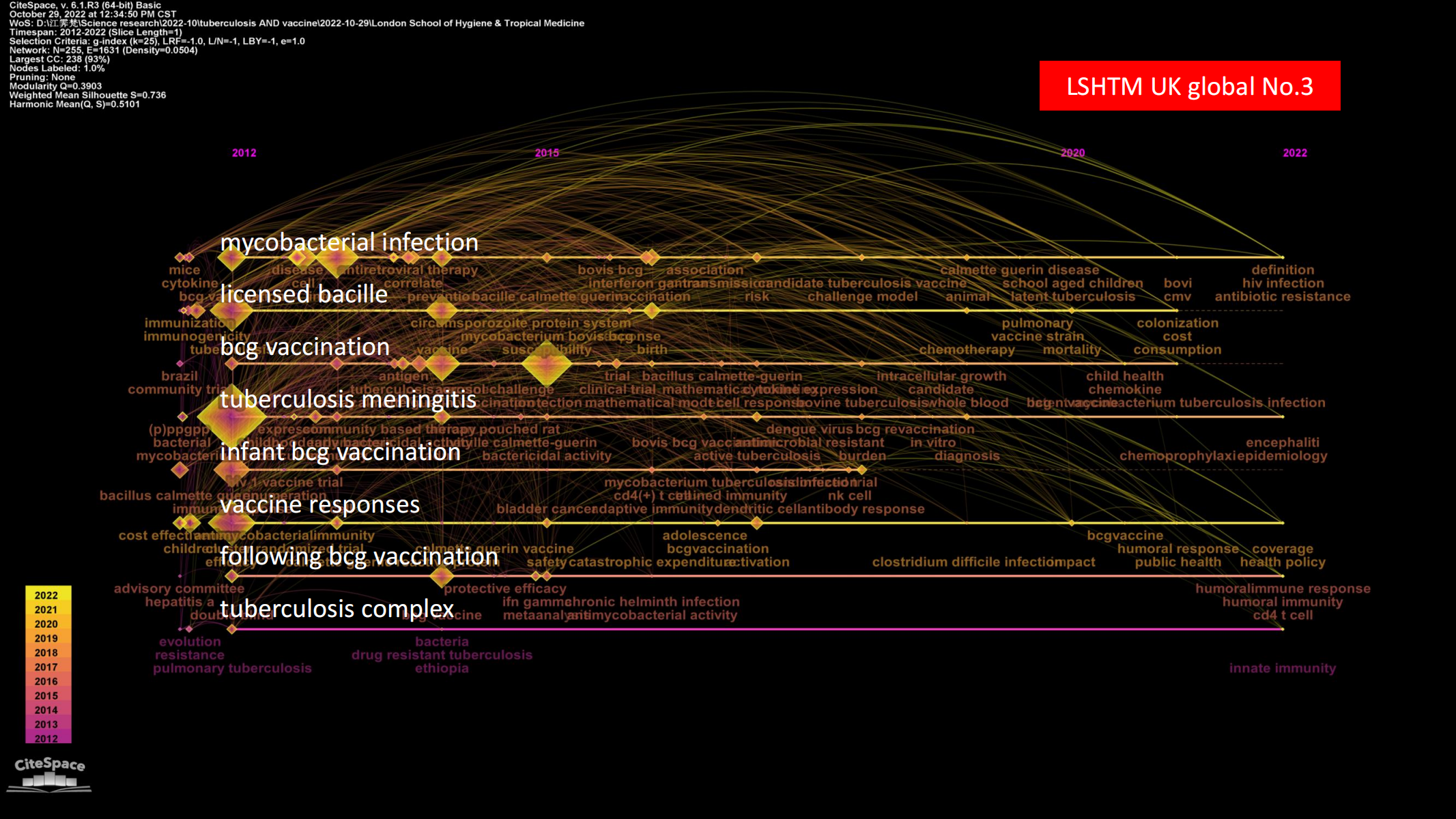

Supplement: Supplementary file 1 [file jpm-13-00408-s001.zip › Supplementary material/Figure S12.png]

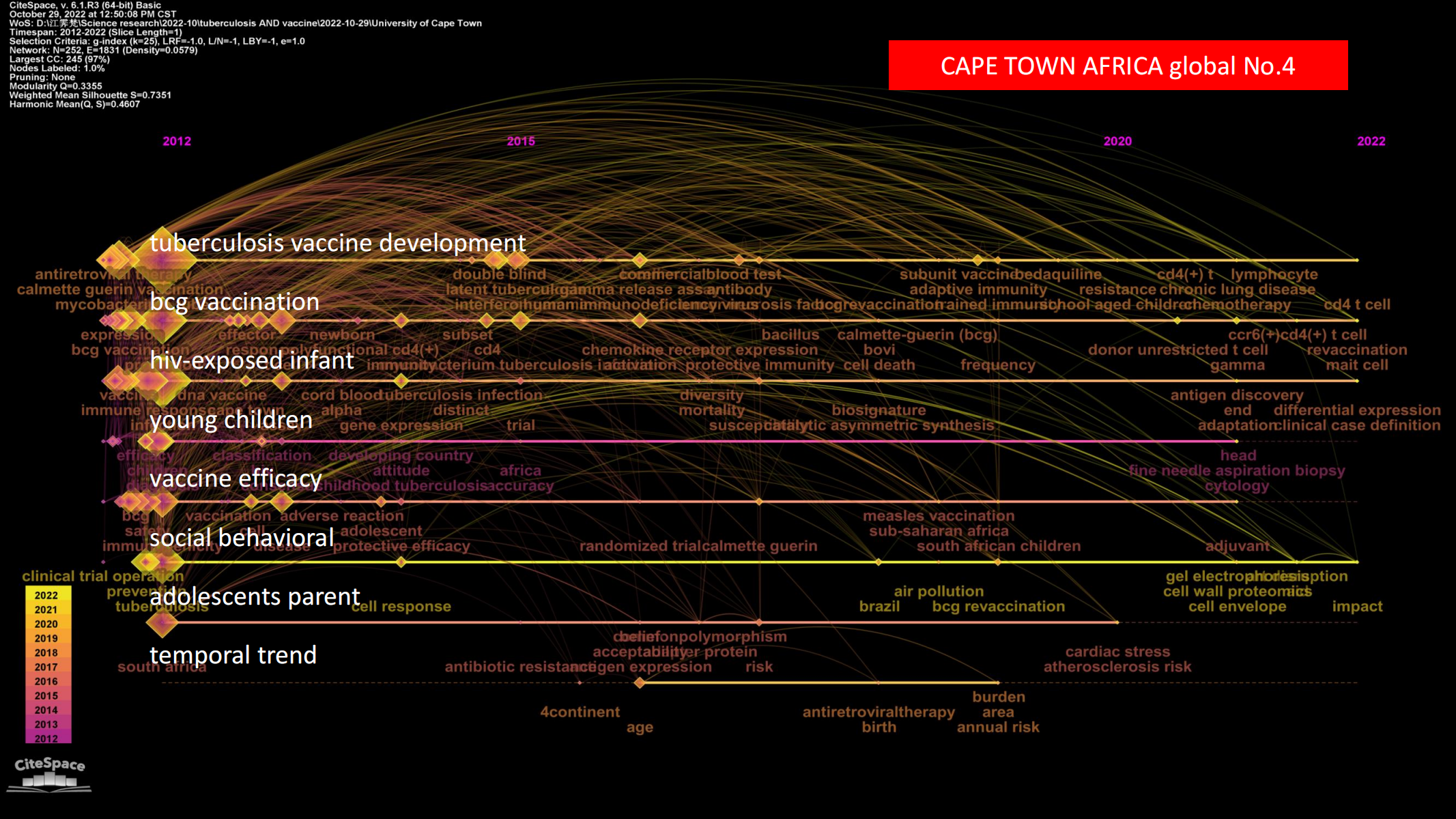

Supplement: Supplementary file 1 [file jpm-13-00408-s001.zip › Supplementary material/Figure S13.png]

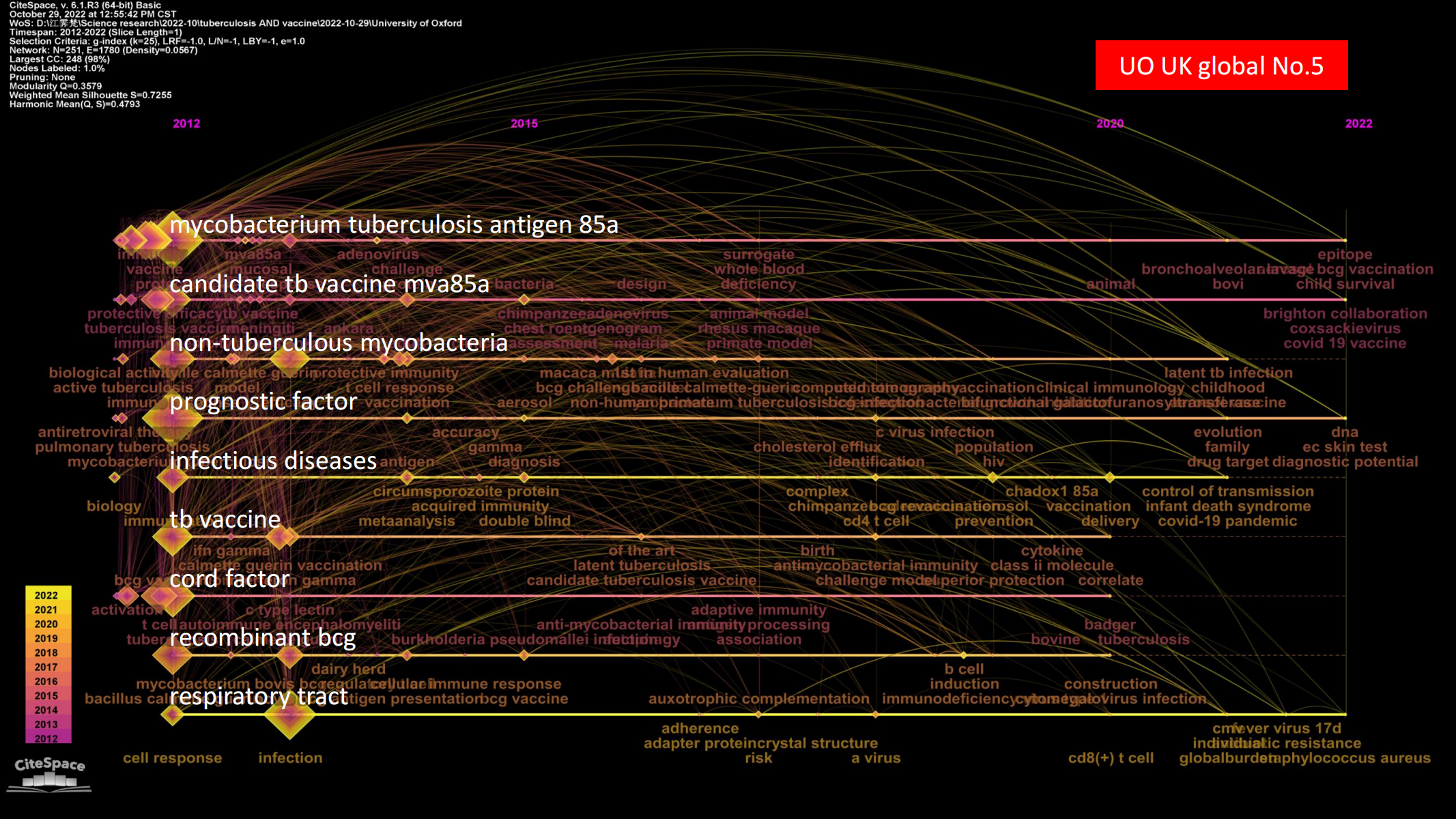

Supplement: Supplementary file 1 [file jpm-13-00408-s001.zip › Supplementary material/Figure S14.png]

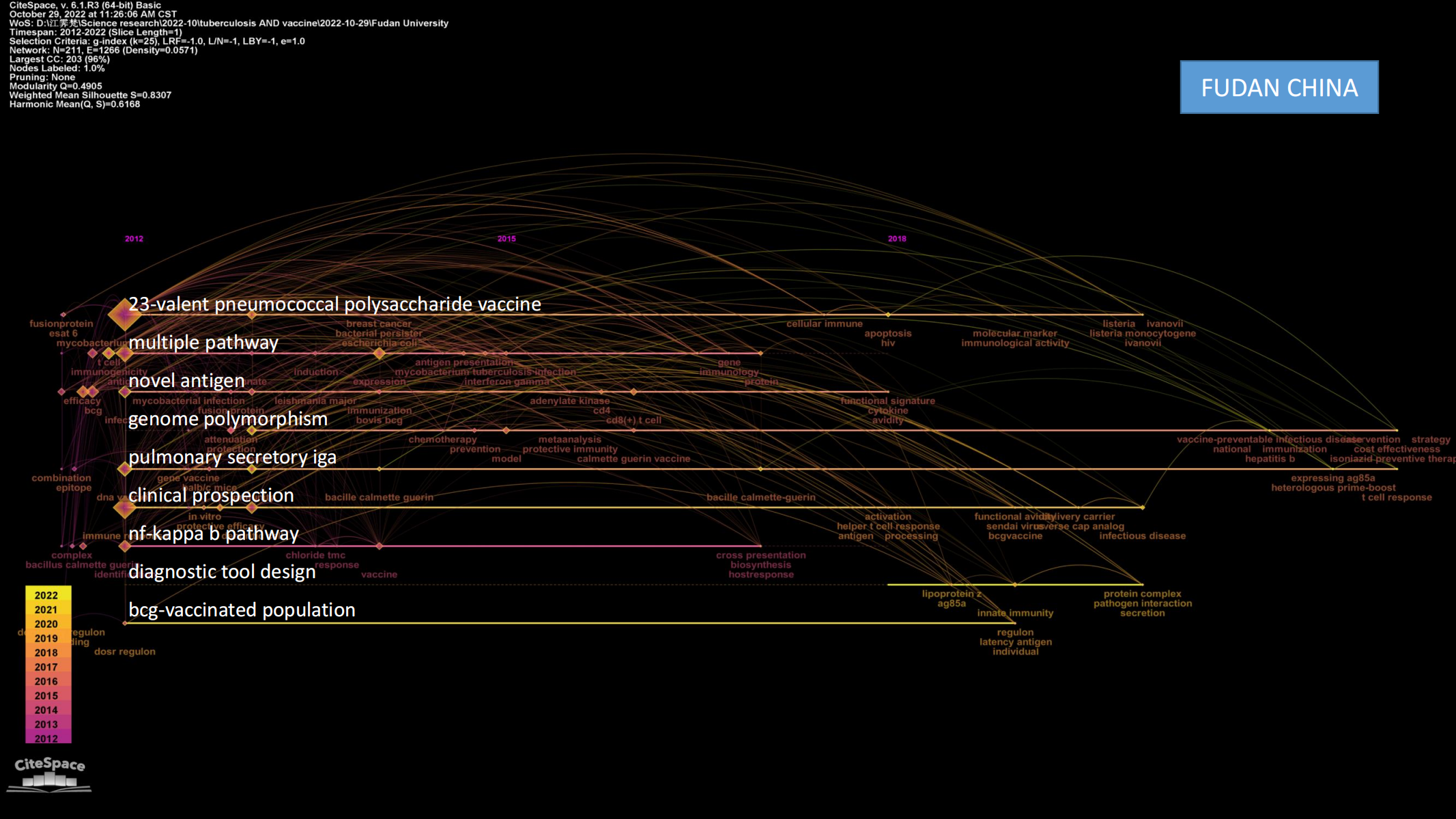

Supplement: Supplementary file 1 [file jpm-13-00408-s001.zip › Supplementary material/Figure S15.png]

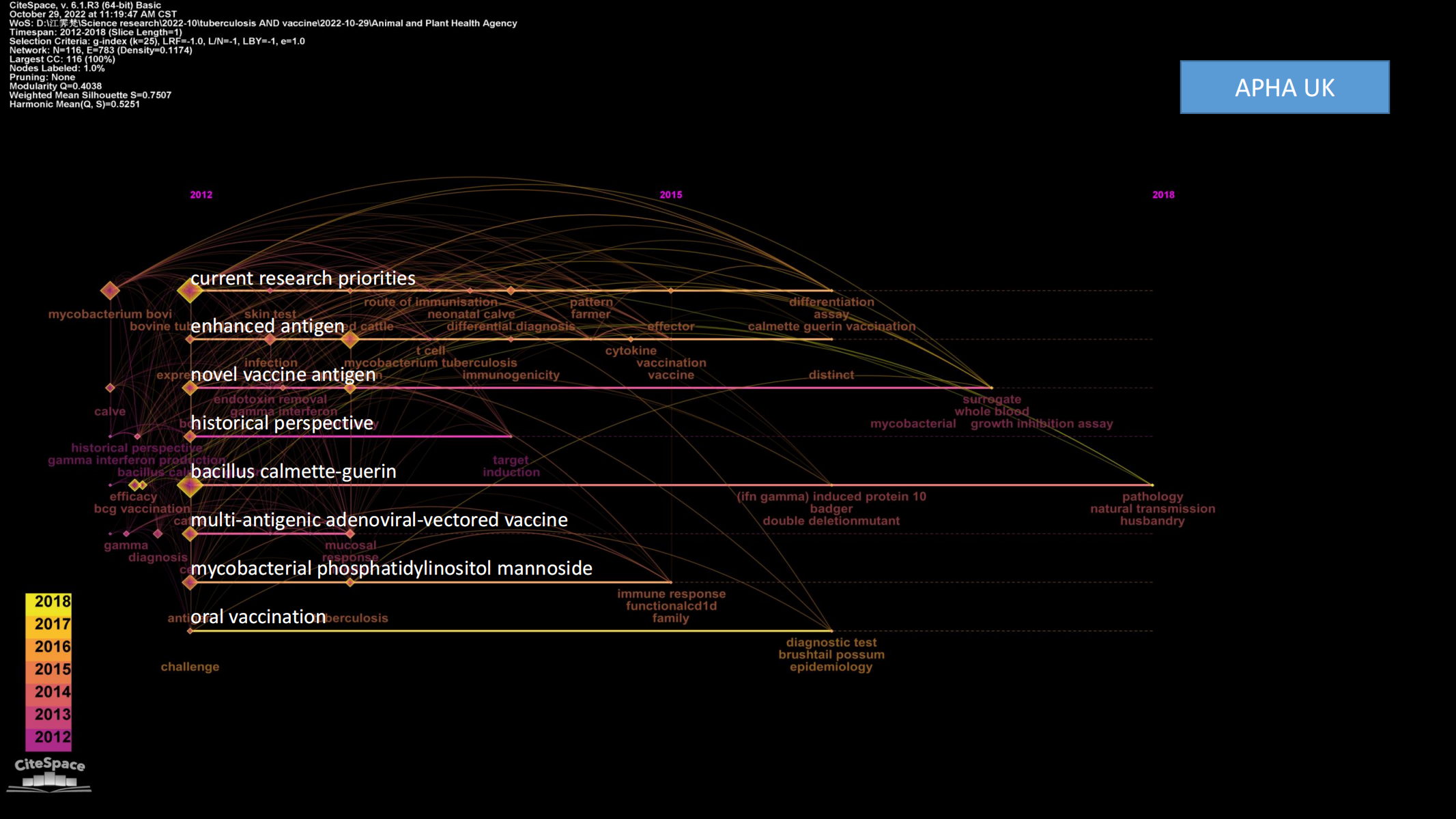

Supplement: Supplementary file 1 [file jpm-13-00408-s001.zip › Supplementary material/Figure S16.png]

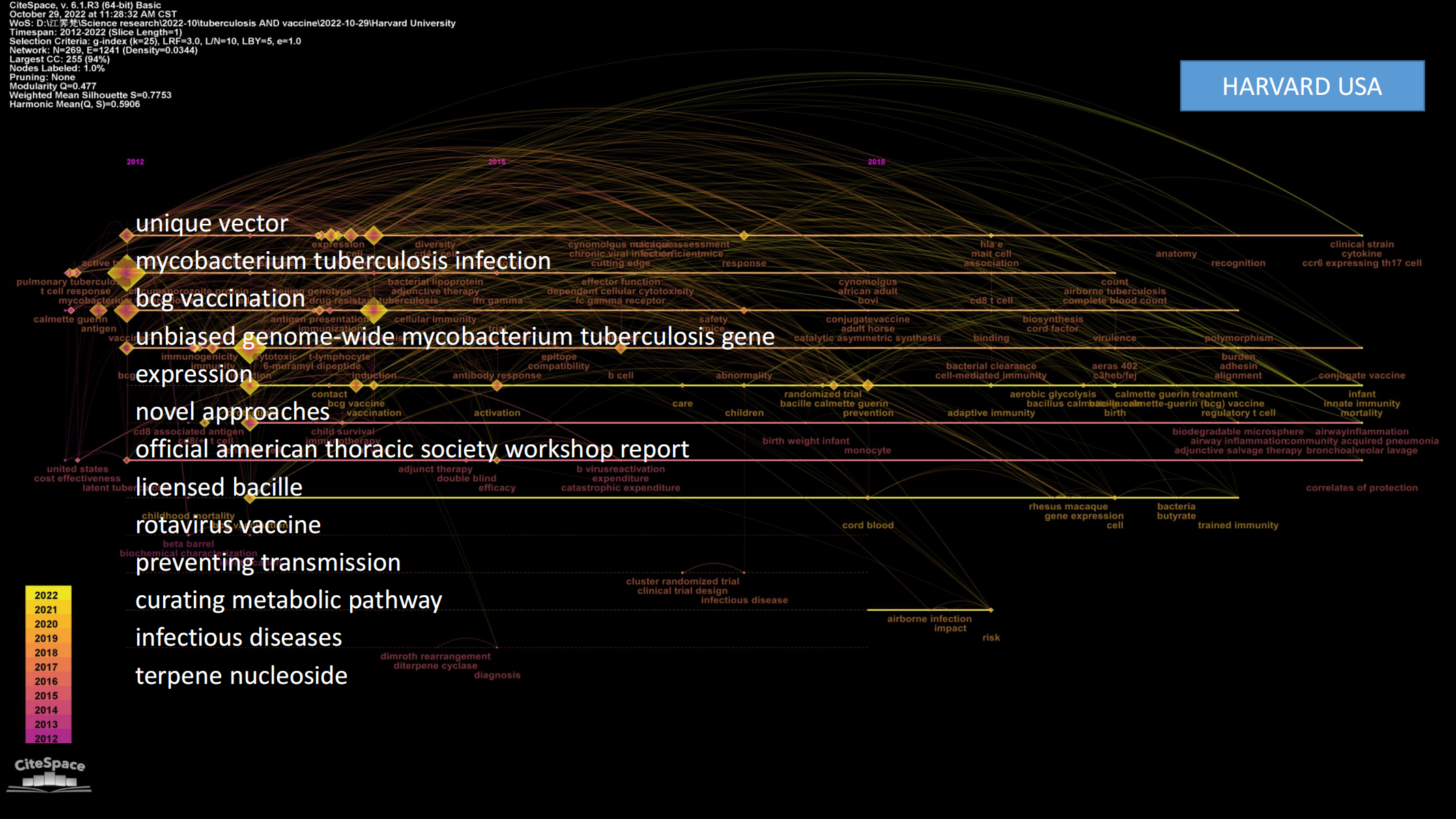

Supplement: Supplementary file 1 [file jpm-13-00408-s001.zip › Supplementary material/Figure S17.png]

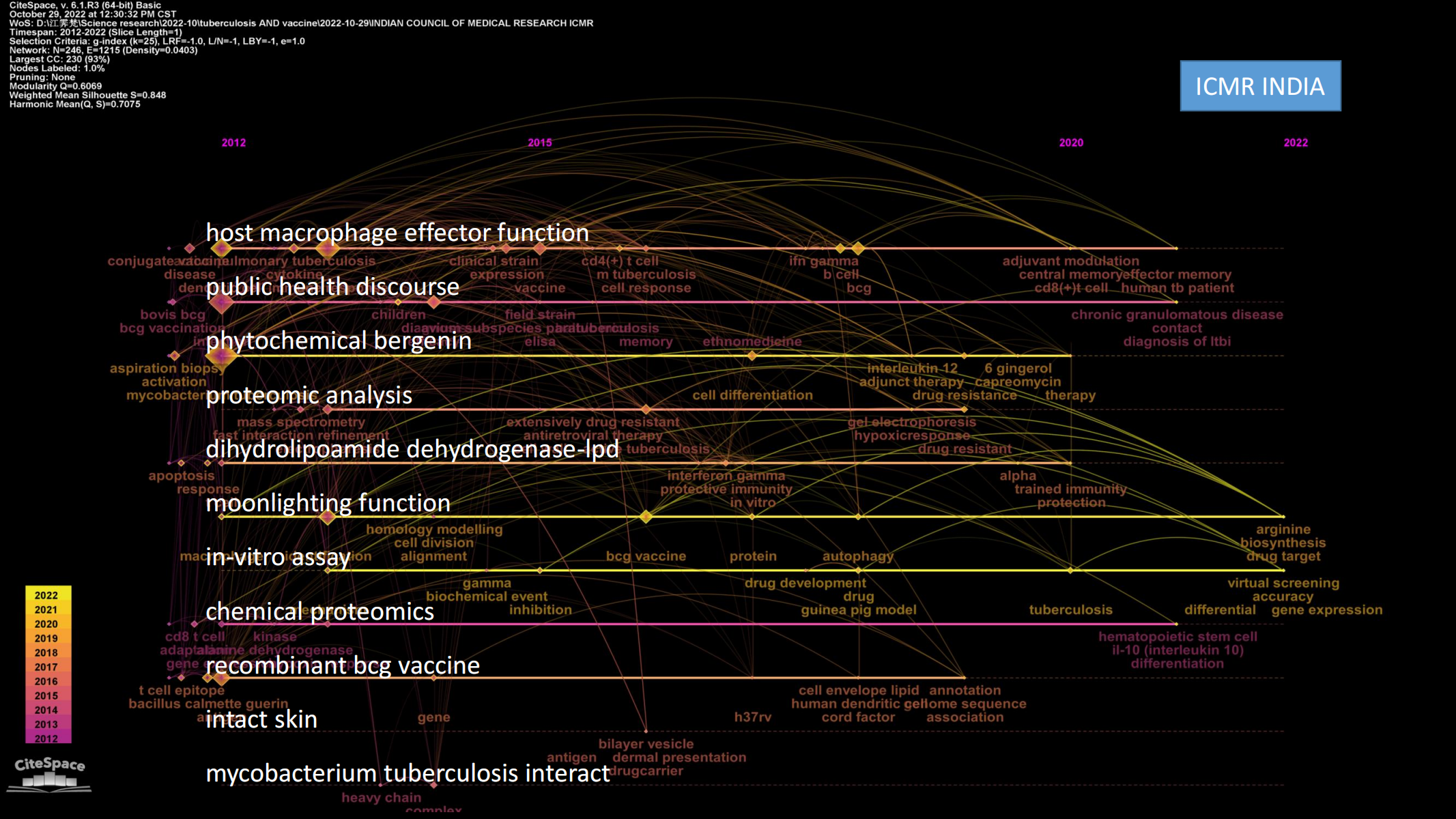

Supplement: Supplementary file 1 [file jpm-13-00408-s001.zip › Supplementary material/Figure S18.png]

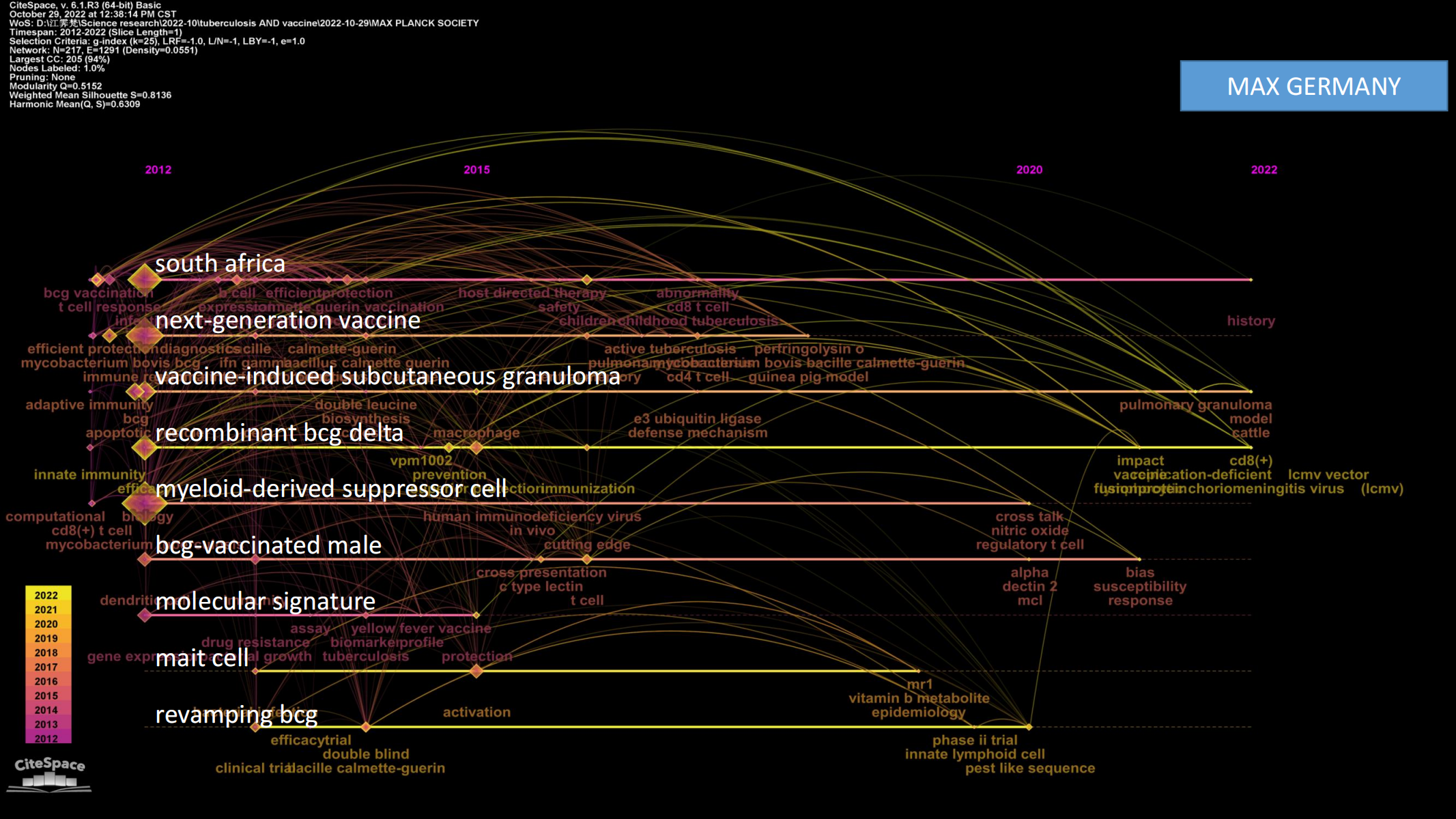

Supplement: Supplementary file 1 [file jpm-13-00408-s001.zip › Supplementary material/Figure S19.png]

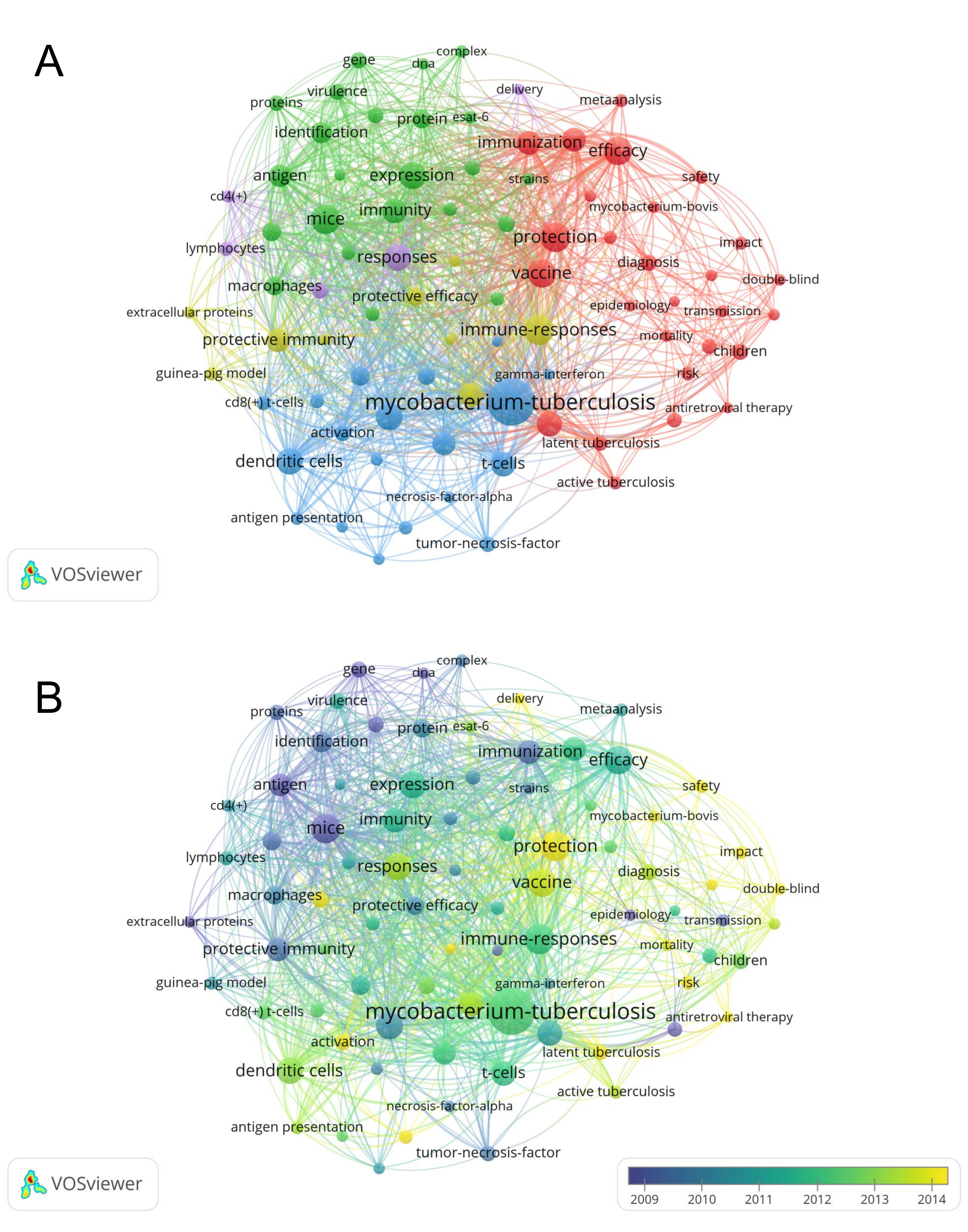

Supplement: Supplementary file 1 [file jpm-13-00408-s001.zip › Supplementary material/Figure S2.png]

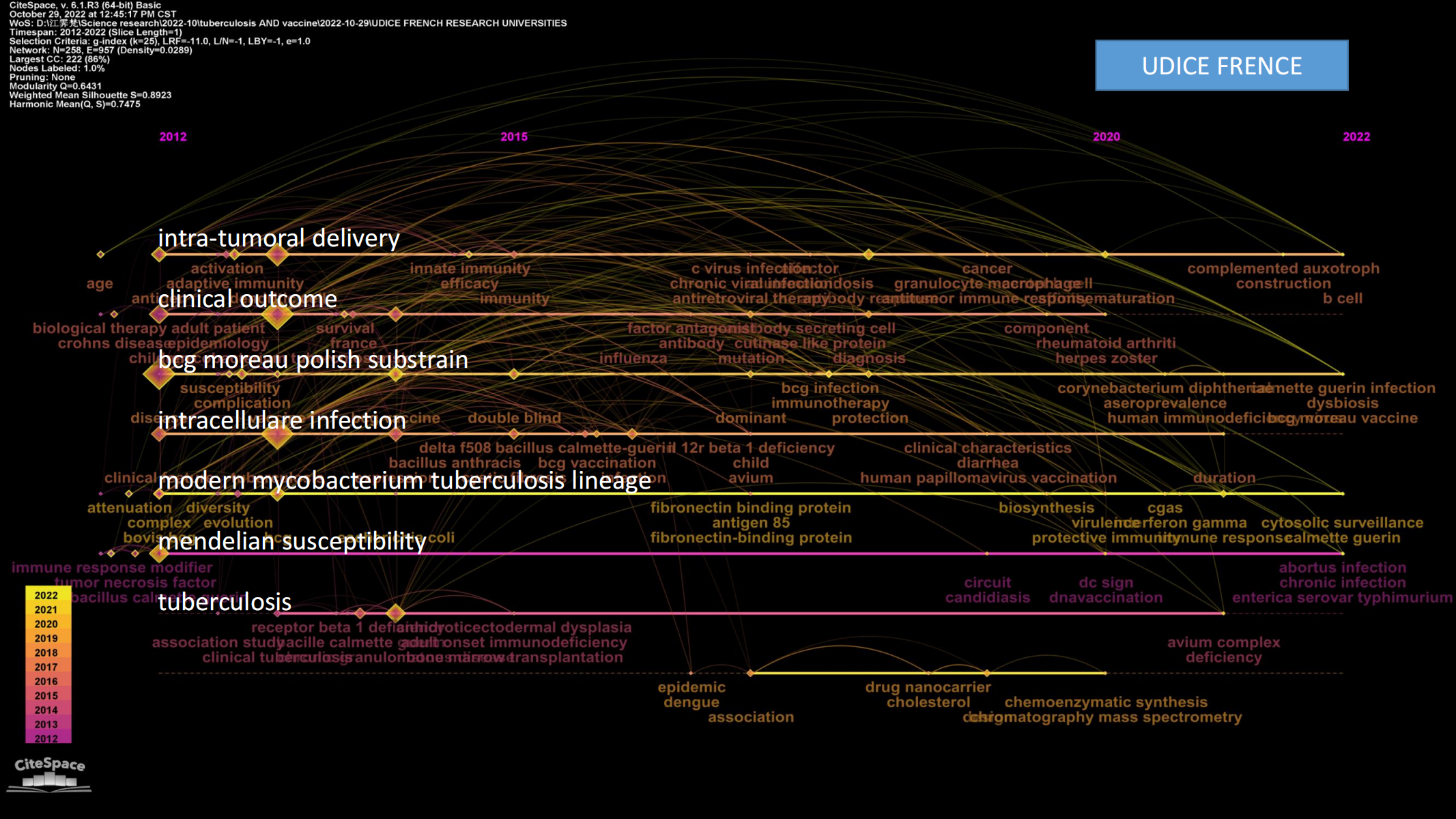

Supplement: Supplementary file 1 [file jpm-13-00408-s001.zip › Supplementary material/Figure S20.png]

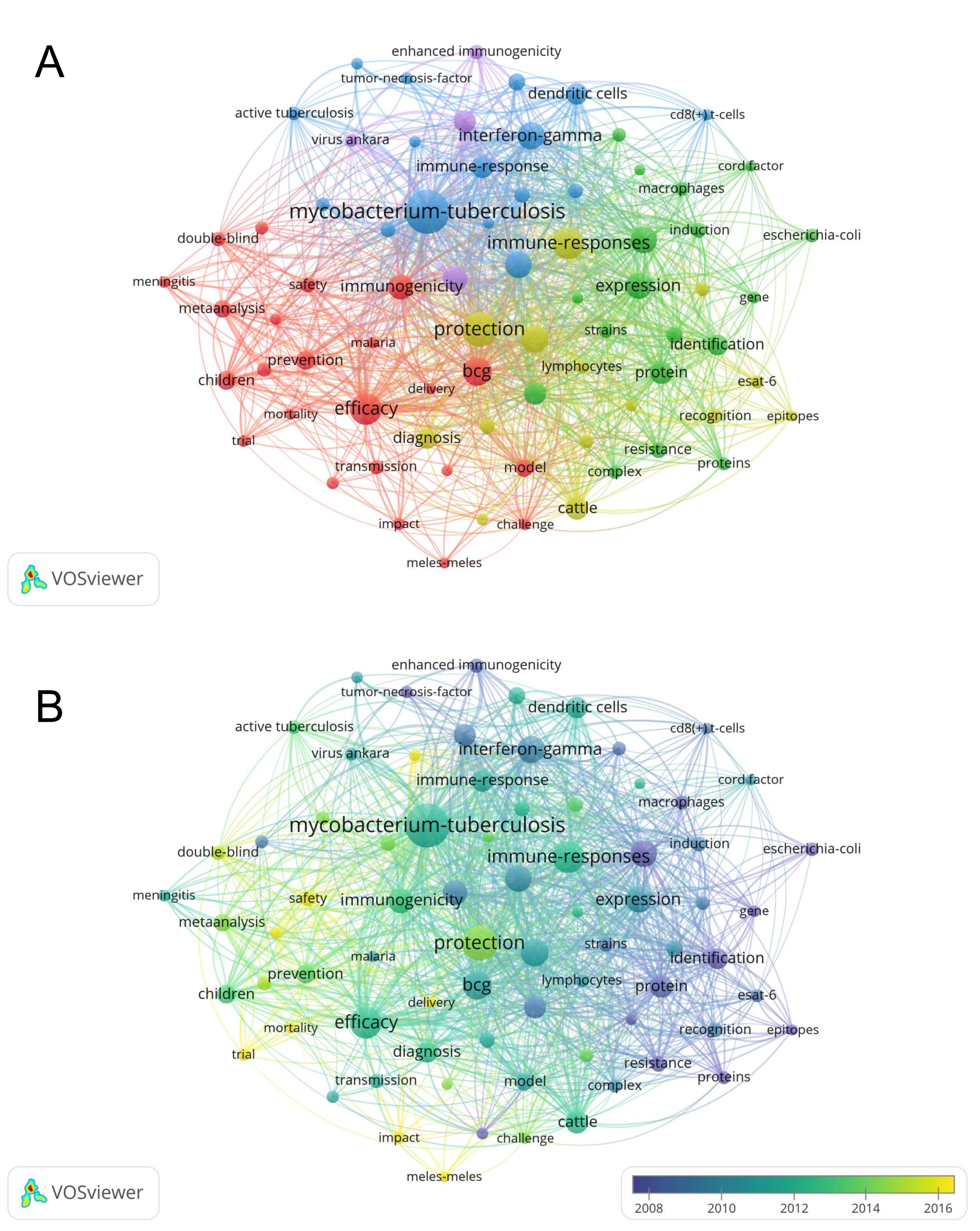

Supplement: Supplementary file 1 [file jpm-13-00408-s001.zip › Supplementary material/Figure S3.png]

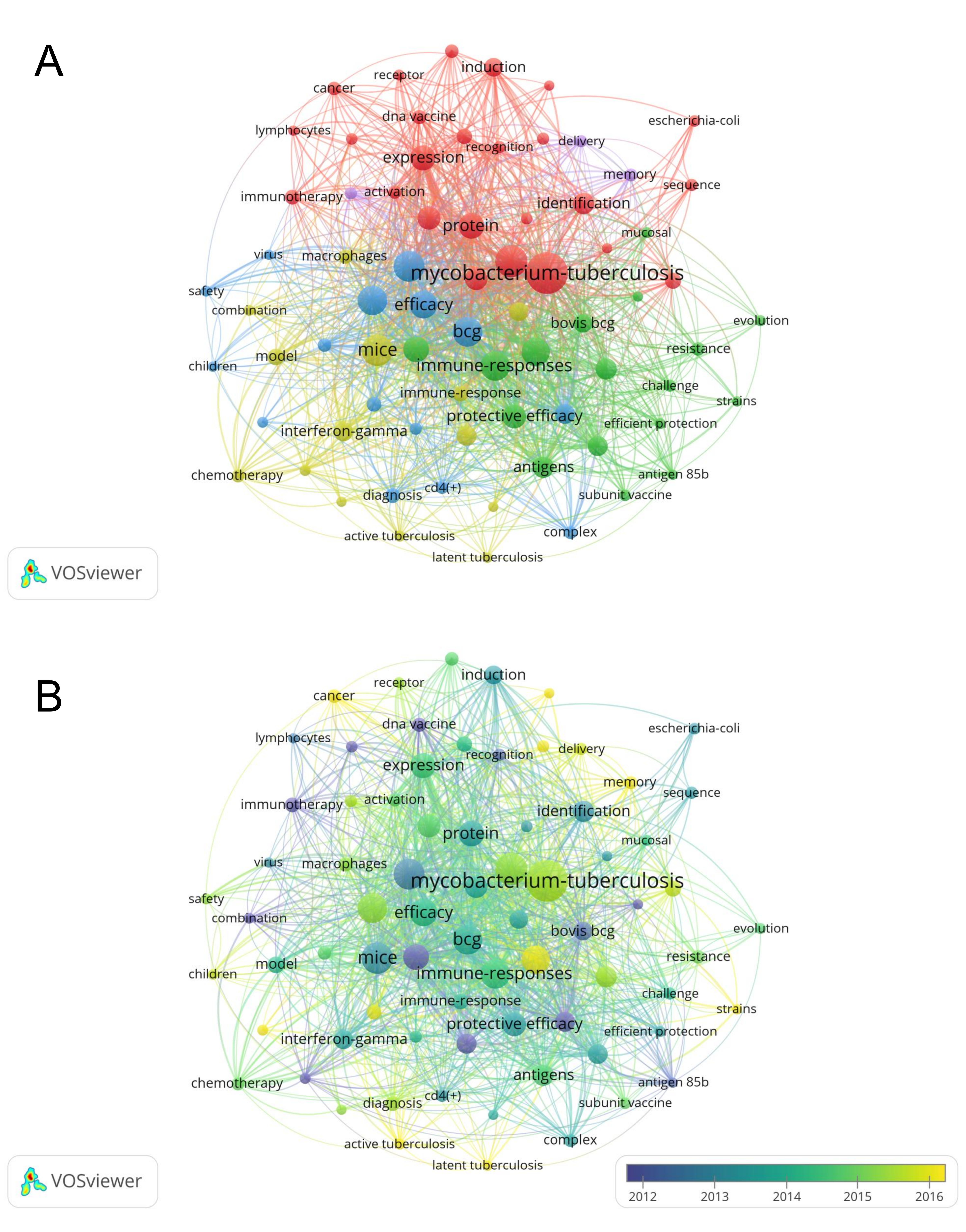

Supplement: Supplementary file 1 [file jpm-13-00408-s001.zip › Supplementary material/Figure S4.png]

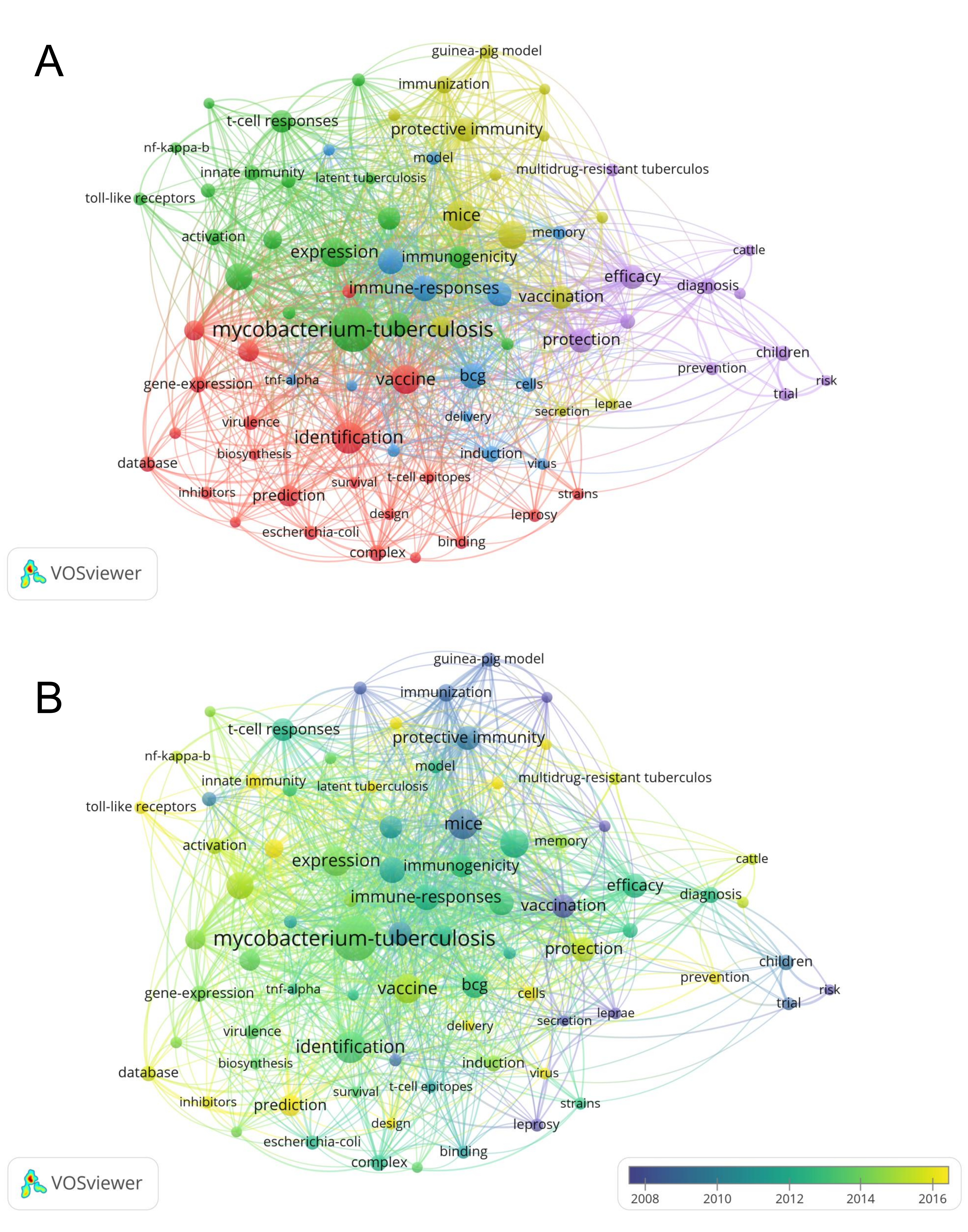

Supplement: Supplementary file 1 [file jpm-13-00408-s001.zip › Supplementary material/Figure S5.png]

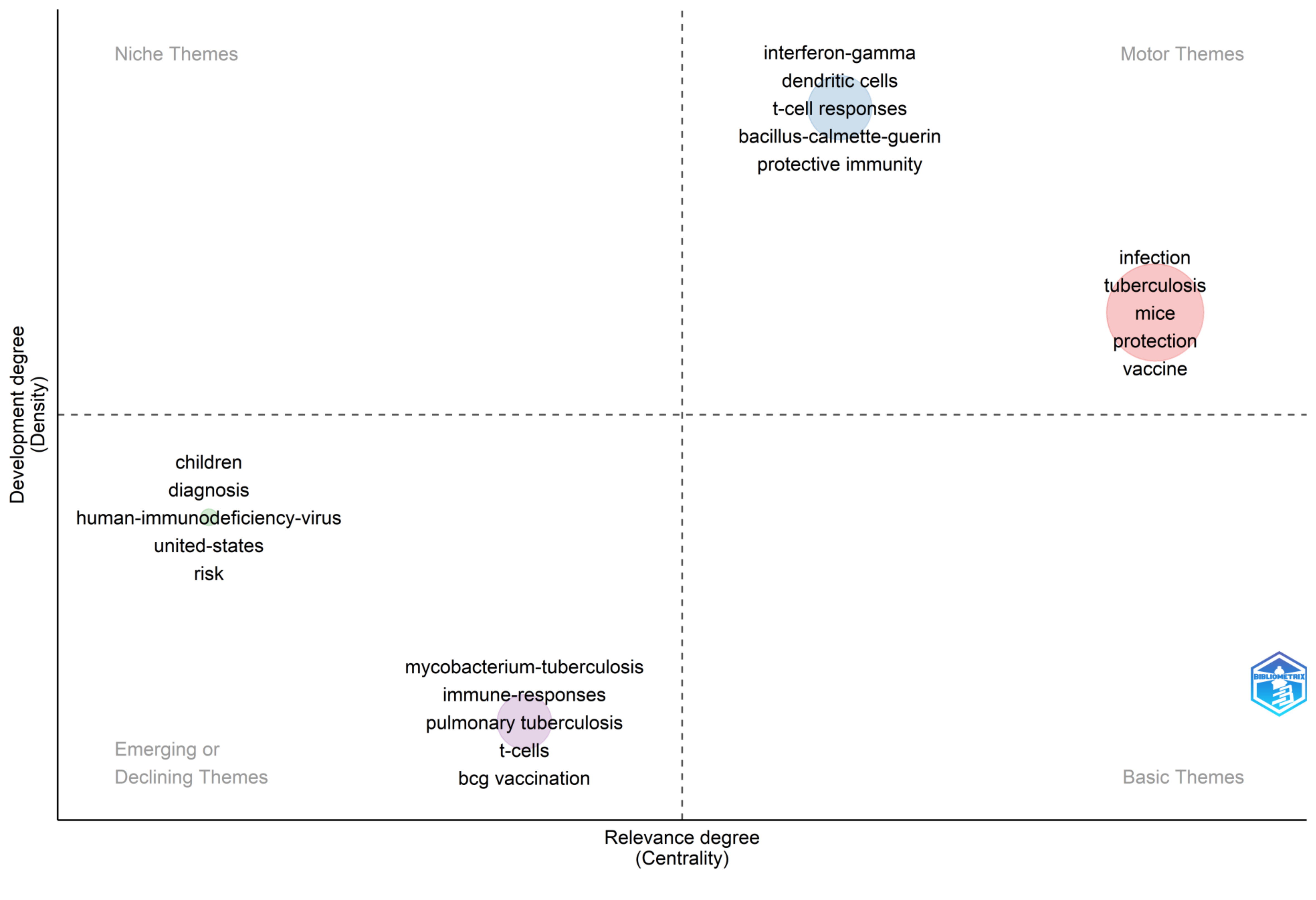

Supplement: Supplementary file 1 [file jpm-13-00408-s001.zip › Supplementary material/Figure S6.png]

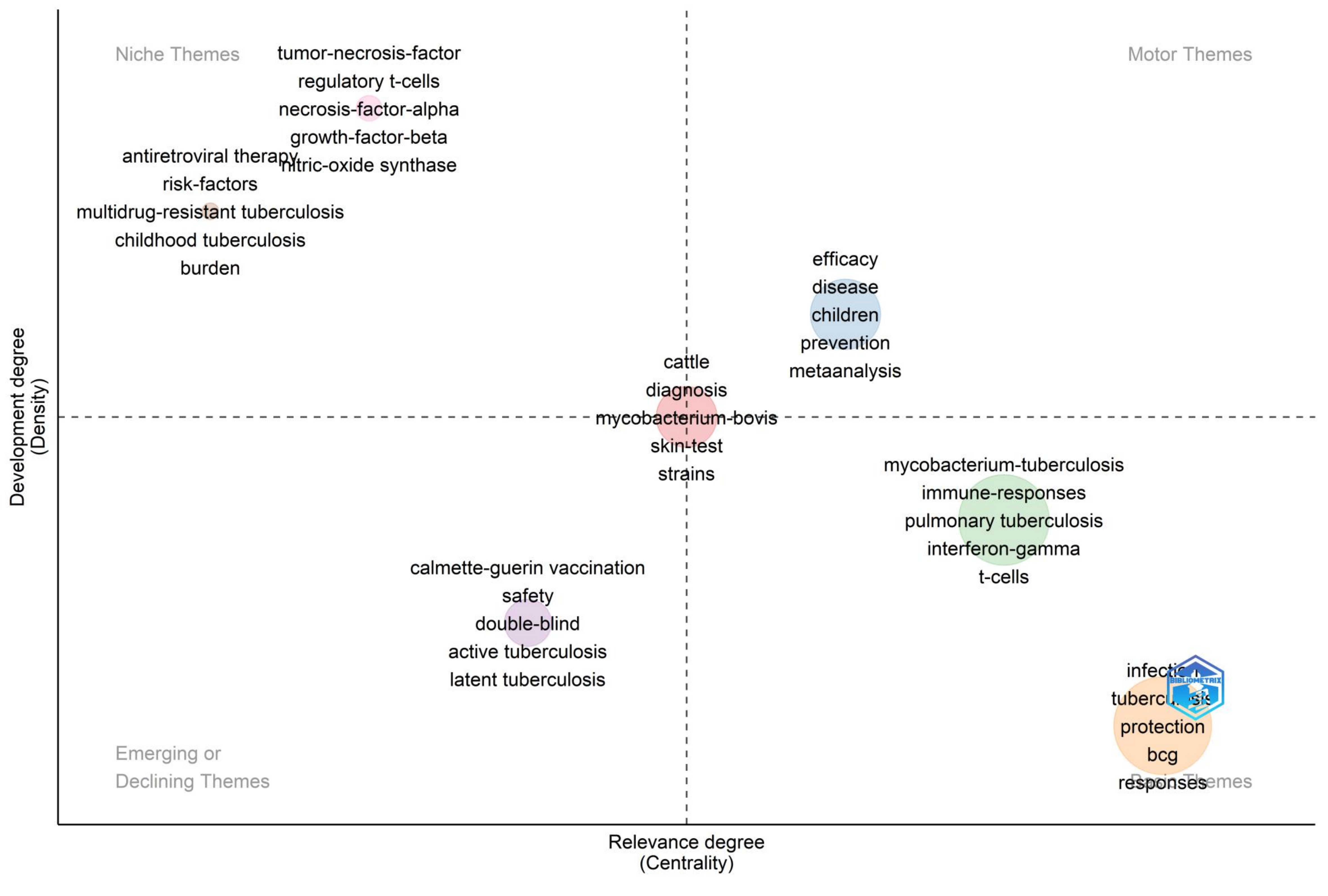

Supplement: Supplementary file 1 [file jpm-13-00408-s001.zip › Supplementary material/Figure S7.png]

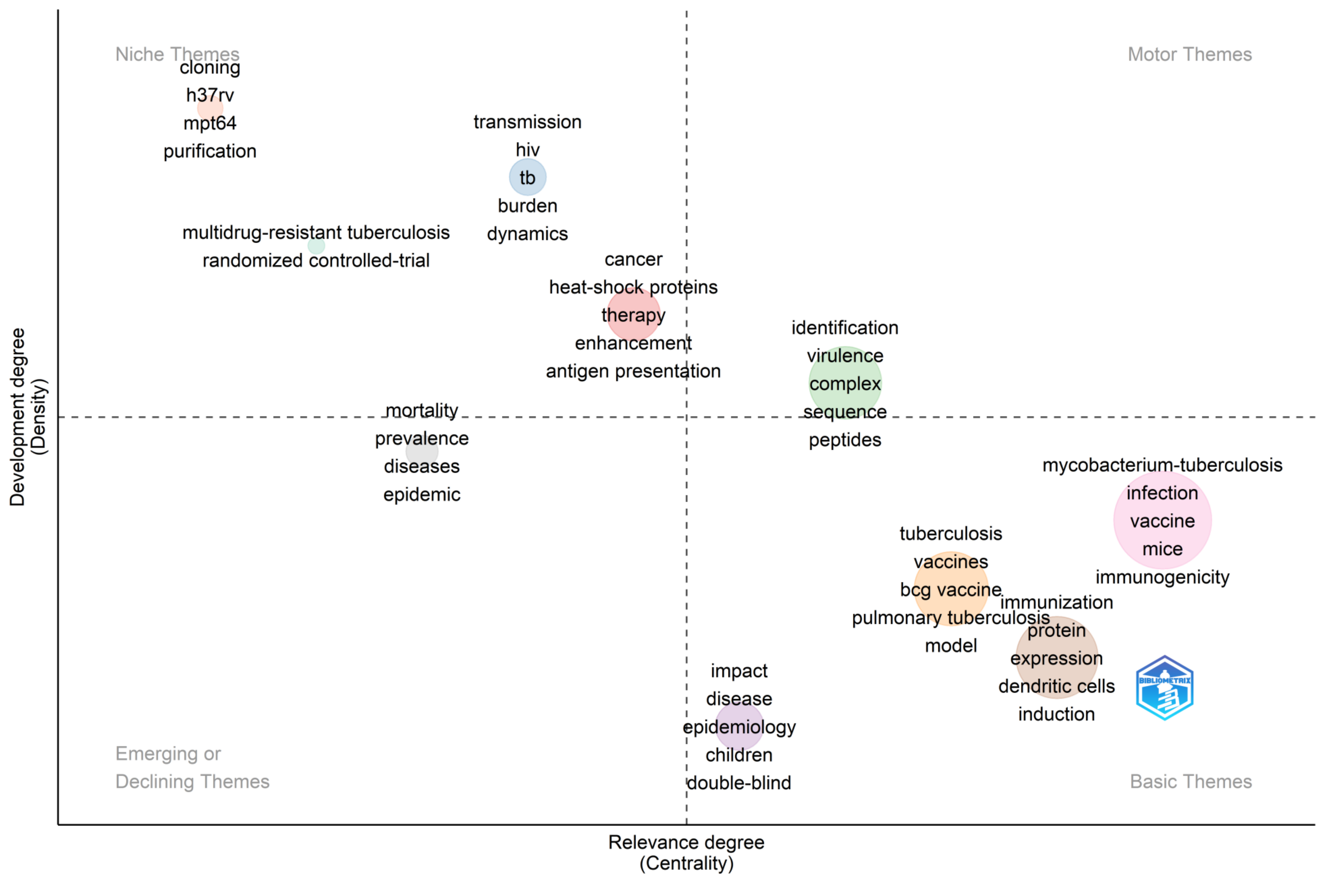

Supplement: Supplementary file 1 [file jpm-13-00408-s001.zip › Supplementary material/Figure S8.png]

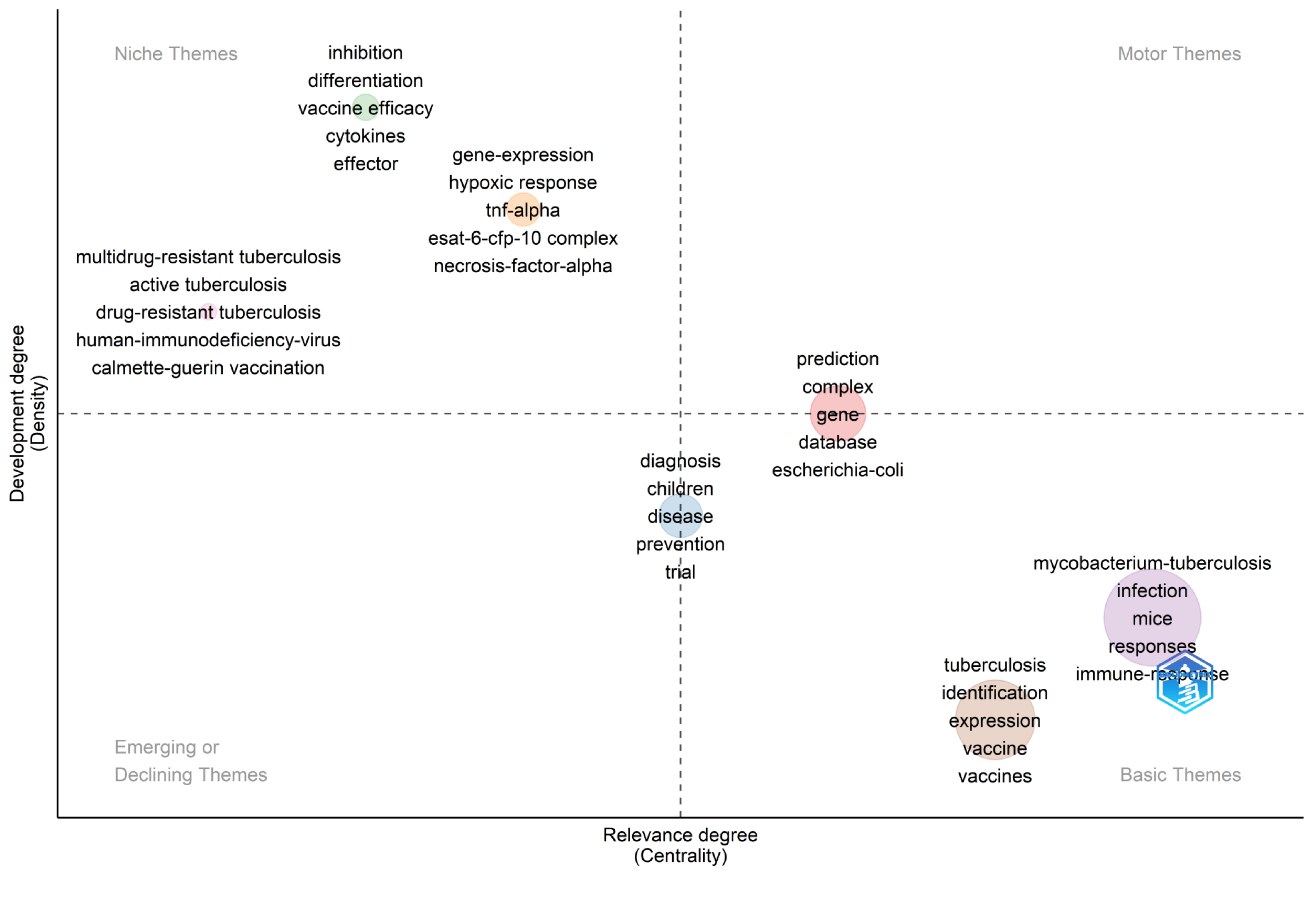

Supplement: Supplementary file 1 [file jpm-13-00408-s001.zip › Supplementary material/Figure S9.png]
